# Supplementary material for: Dual Targeting of PDGFRα and FGFR1 Displays Synergistic Efficacy in Malignant Rhabdoid Tumors
Source: Cell Rep. 2016 Oct 25;17(5):1265–75. doi: 10.1016/j.celrep.2016.10.005 (PMC5098123; doi:10.1016/j.celrep.2016.10.005)
Supplement: Document S1. Supplemental Experimental Procedures, Figures S1–S4, and Tables S1 and S2 [file mmc1.pdf]

Cell Reports, Volume *17*

# Supplemental Information

# Dual Targeting of PDGFR$\alpha$ and FGFR1 Displays Synergistic Efficacy in Malignant Rhabdoid Tumors

**Jocelyn P. Wong, Jason R. Todd, Martina A. Finetti, Frank McCarthy, Malgorzata Broncel, Simon Vyse, Maciej T. Luczynski, Stephen Crosier, Karen A. Ryall, Kate Holmes, Leo S. Payne, Frances Daley, Patty Wai, Andrew Jenks, Barbara Tanos, Aik-Choon Tan, Rachael C. Natrajan, Daniel Williamson, and Paul H. Huang**

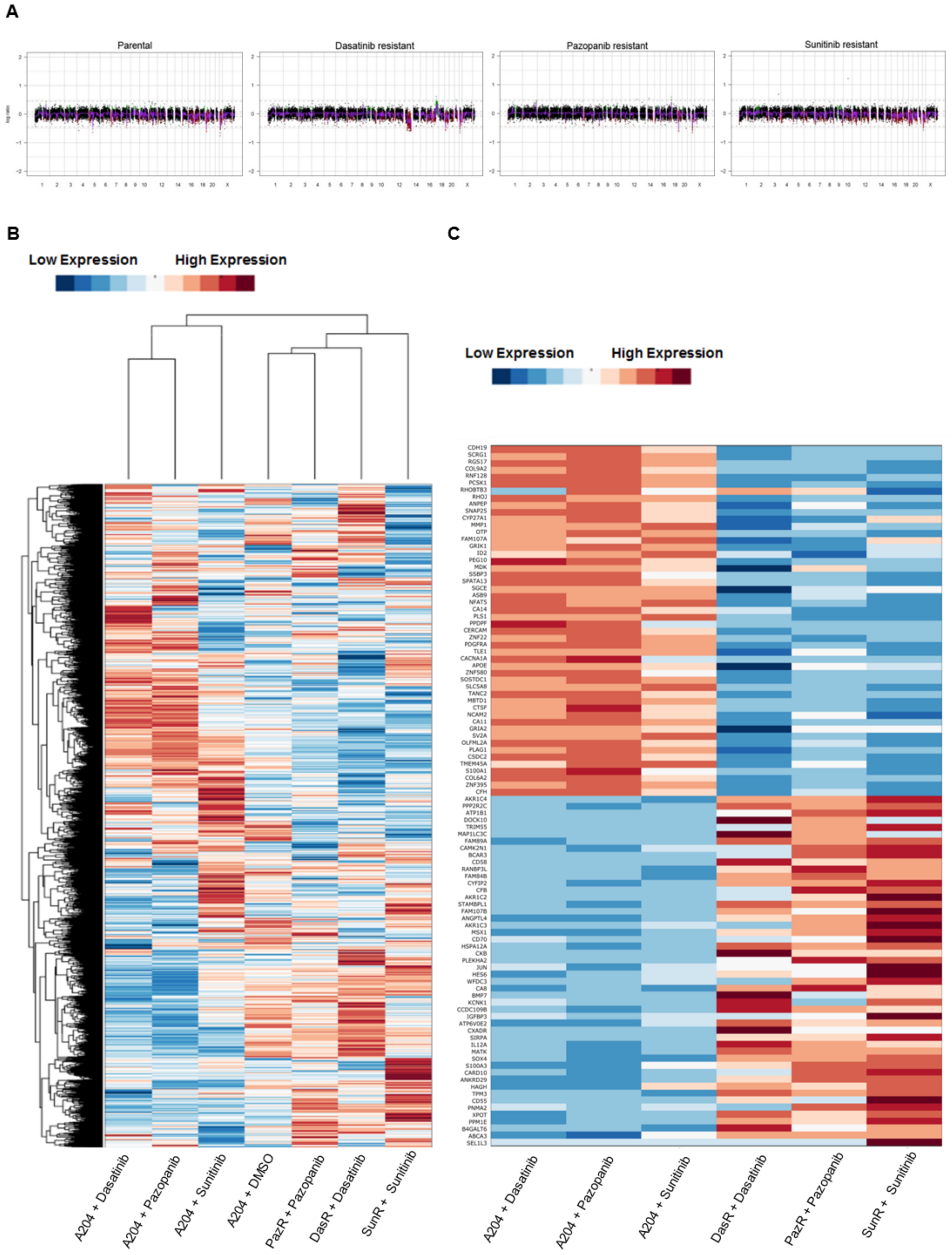


**Figure S1.** ***Related to Figure 2*** (A) Microarray-based comparative genomic hybridisation plots of A204 parental and resistant cells displaying the full genomic profiles of the four cell lines. (B) Hierarchical clustering of gene expression dataset of parental A204 cells treated with DMSO control or each of the three PDGFRα TKIs and each resistant subline treated with their respective TKI. DasR=dasatinib resistant, PazR=pazopanib resistant and SunR=sunitinib resistant. (C) Heatmap of the top 50 upregulated and downregulated genes in the resistant sublines versus the parental A204 cells treated with TKIs.

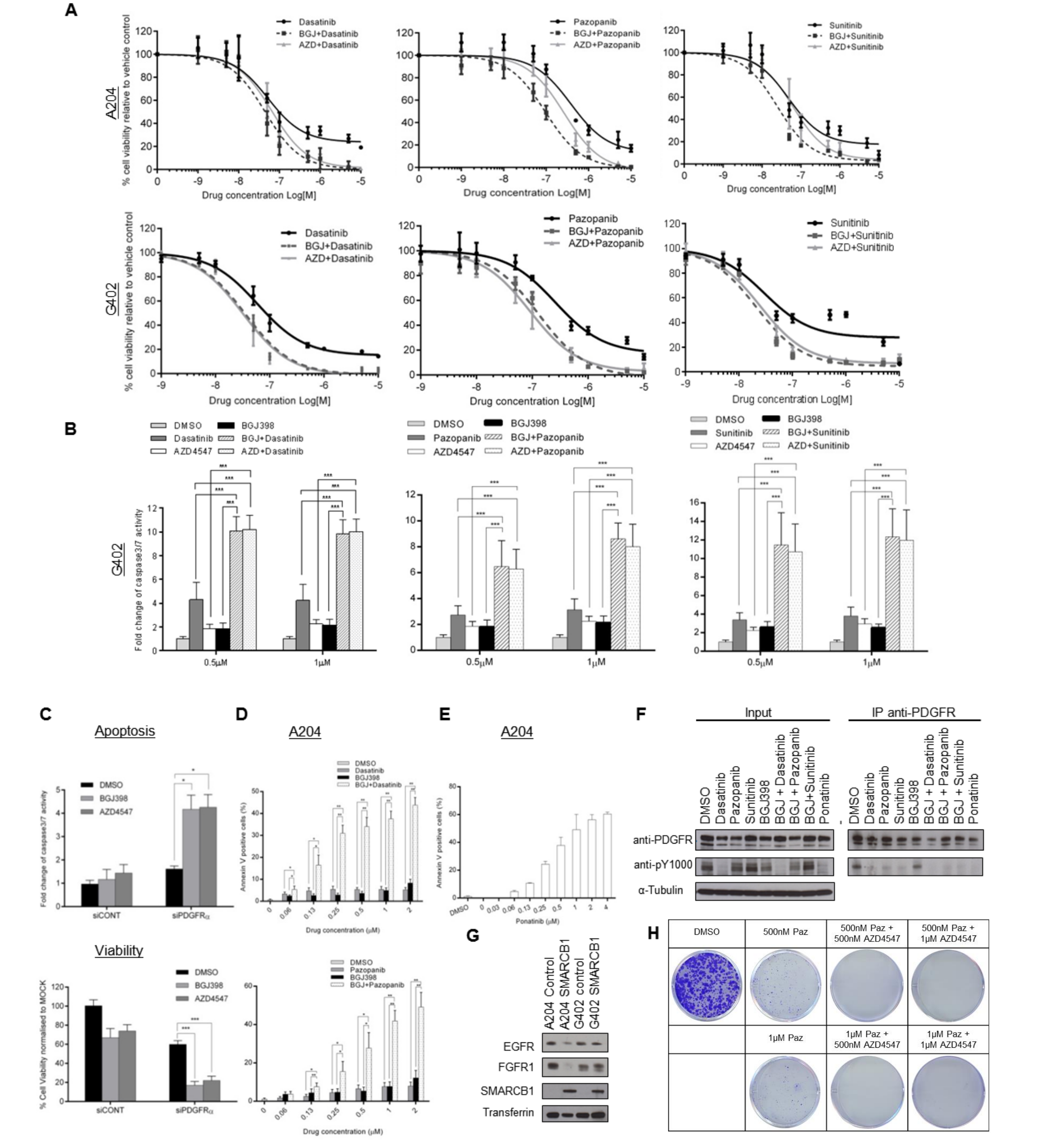

**Figure S2. *Related to Figure 3*** Dual inhibition of PDGFRα and FGFR1 is cytotoxic in MRT cells. (A) Dose response curves for A204 and G402 cells upon treatment with PDGFRα and a combination of PDGFRα and FGFR inhibitors. Cell viability data is normalised to DMSO control (n=3). (B) Bar plots showing the normalised fold change in caspase 3/7 activity in the G402 cells upon treatment with PDGFRα and FGFR inhibitors or a combination at the indicated doses (n=3). Data is normalised to DMSO control. Statistical significance of combination versus single TKI treatment was performed by ANOVA with Tukey's multiple comparisons test where ***$p<0.001$. (C) Bar plots showing apoptosis measured by caspase 3/7 activity (upper panel) and viability (lower panel) of A204 cells treated with FGFR inhibitors in combination with siRNA depletion of PDGFRα. Statistical analysis of FGFR inhibitors versus DMSO control was performed by paired Student's t test where *$p<0.05$ and ***$p<0.001$. (D) Bar plots showing percentage Annexin V staining in A204 parental cells when treated with PDGFRα inhibitor, BGJ398 or a combination of both inhibitors (n=3) where *$p<0.05$ and **$p<0.01$. (E) Bar plots showing percentage Annexin V staining in A204 parental cells treated with ponatinib (n=3). (F) Immunoprecipitation of PDGFRα followed by immunoblotting with phosphotyrosine-specific antibody (PY1000) in A204 cells upon treatment with 1μM PDGFRα inhibitor, BGJ398, combination or ponatinib for 1 hour. (G) Immunoblot of FGFR1 and EGFR showing modulation of receptor levels upon ectopic SMARCB1 expression in A204 and G402 cells. (H) Colony formation assay showing that pazopanib treatment over 2 weeks leads to resistant colony formation in the A204 cells. However treatment with high dose combination of pazopanib and AZD4547 led to no colonies, providing support that first line combination therapy prevents acquisition of resistance. For (A), (B), (C), (D) and (E), all values are mean ± SD.

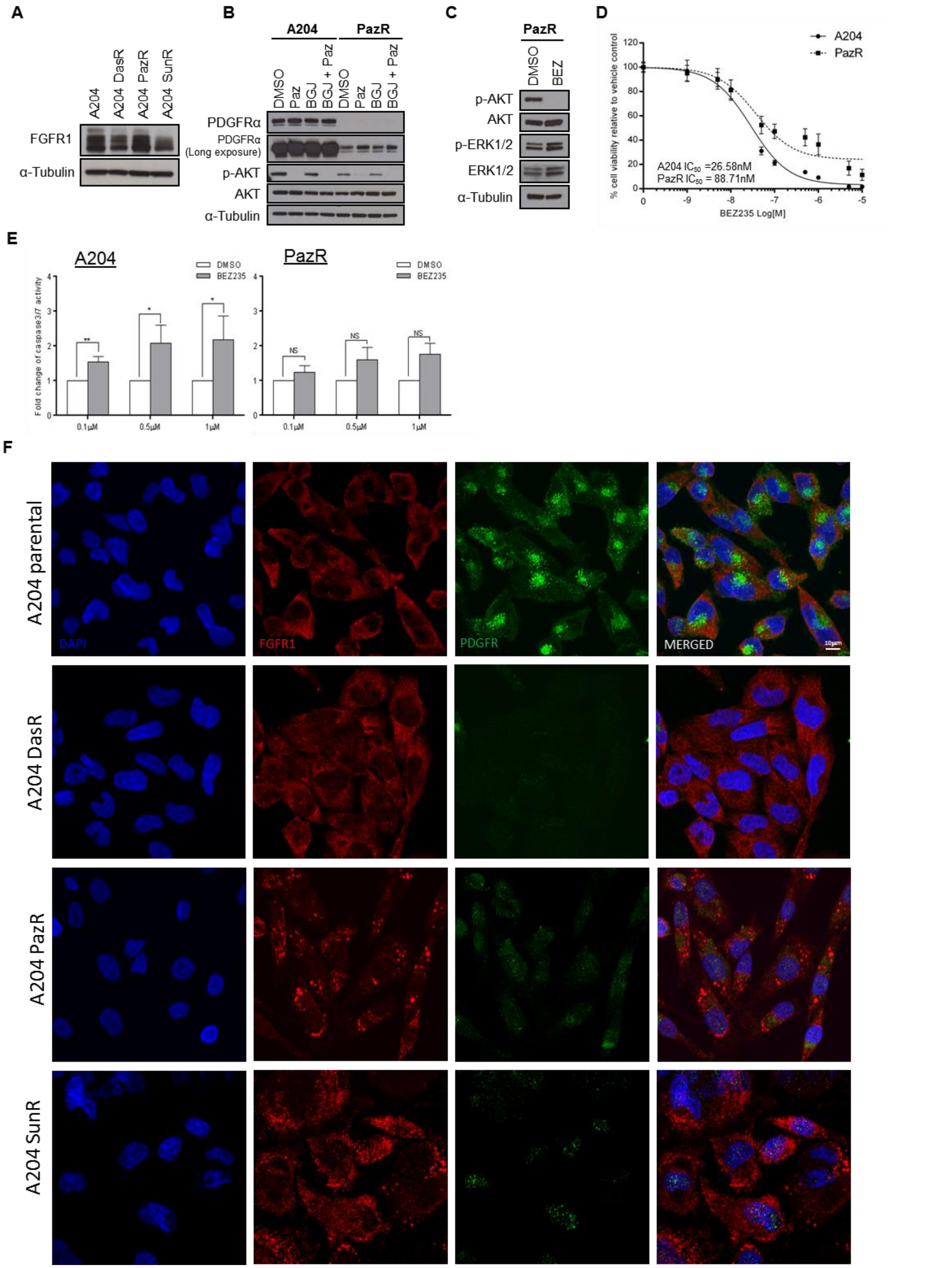


**Figure S3.** ***Related to Figure 3*** Targeting FGFR1 sensitizes acquired resistance to pazopanib. (A) Immunoblot of FGFR1 expression in the parental A204 and resistant sublines. DasR=dasatinib resistant, PazR=pazopanib resistant and SunR=sunitinib resistant. (B) Immunoblot of PDGFRα levels and AKT pathway activation in the parental A204 and PazR resistant cells upon treatment with 1μM pazopanib, BGJ398 or a combination for 1 hour. (C) Immunoblot of AKT and ERK1/2 phosphorylation levels in PazR cells upon treatment with 1μM BEZ235 for 1 hour. (D) Dose response curves for A204 and PazR cells upon treatment with BEZ235. Cell viability data is normalised to DMSO control (n=3). Values are mean ± SD. (E) Bar plots showing the normalised fold change in caspase 3/7 activity in the A204 (left) and PazR (right) cells upon treatment with BEZ235 at the indicated doses (n=3). Data is normalised to DMSO control. Statistical significance of DMSO versus BEZ235 treatment was performed by paired Student's t test where $*p<0.05$ and NS is not significant. (F) Representative images of dual-colour immunofluorescence analysis of parental A204 and resistant sublines, DAPI (blue), FGFR1 (red) and PDGFRα (green) showing that FGFR1 and PDGFRα expression is uniformly distributed in all cells within the parental A204 population.

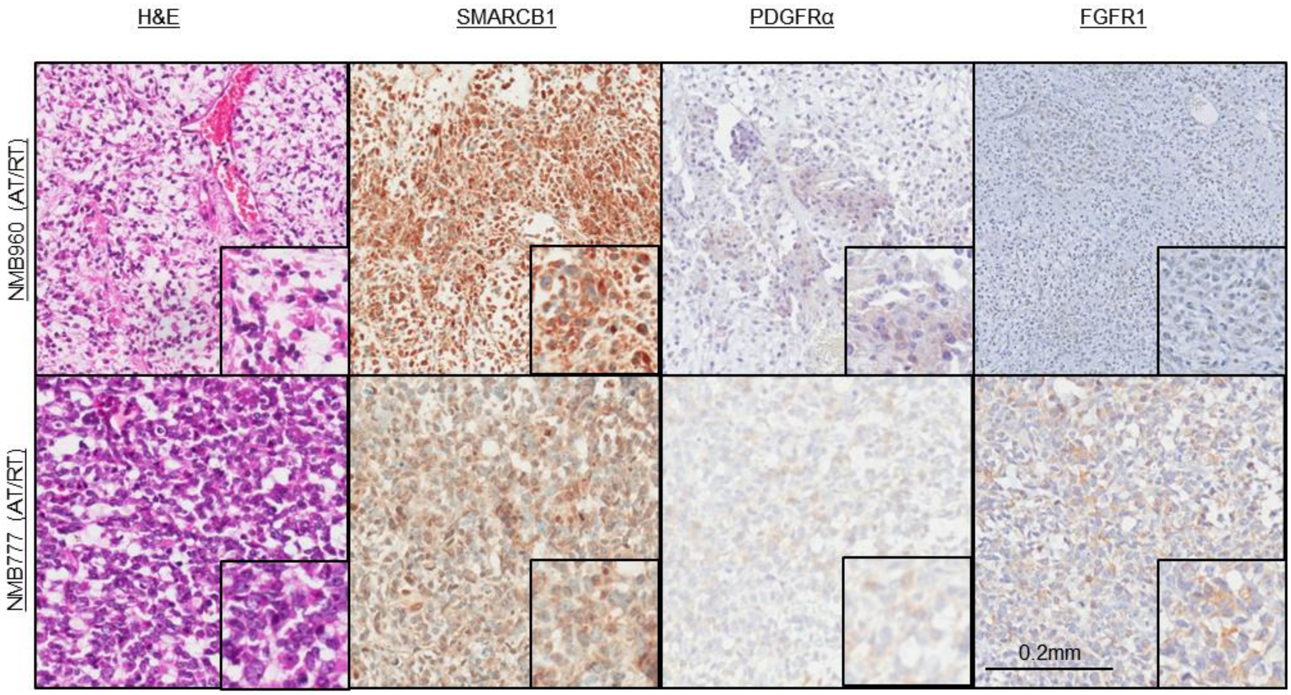


**Figure S4.** ***Related to Figure 4*** Immunohistochemical analysis of AT/RT patient specimens for haematoxylin and eosin (H&E), anti-SMARCB1, anti-PDGFRα and anti-FGFR1 staining. Scale bar represents 0.2mm.

**Table S1. Dasatinib, Pazopanib, Sunitinib IC50 concentrations in a panel of 14 cell lines and A204 resistant cell lines .**
*Related to Figure 1.*

| Cell Line | Dasatinib IC50 (nM) | Pazopanib IC50 (nM) | Sunitinib IC50 (nM) |
|---|---|---|---|
| SAOS2 | 1152.3 +/- 311.0 | >10000 | 4569.7 +/- 516.2 |
| U2OS | >10000 | >10000 | >10000 |
| HT1080 | >10000 | >10000 | >10000 |
| MES-SA | >10000 | >10000 | >10000 |
| SJSA-1 | >10000 | >10000 | >10000 |
| SW684 | 62.4 +/- 25.9 | >10000 | >10000 |
| SW872 | 1038 +/- 490.7 | >10000 | >10000 |
| SW982 | 188.3 +/- 64.7 | >10000 | 581.6 +/- 117.0 |
| Hs729T | >10000 | >10000 | >10000 |
| RMS-YM | >10000 | >10000 | >10000 |
| RUCH-3 | >10000 | >10000 | >10000 |
| T91-95 | >10000 | >10000 | >10000 |
| G402 | 62.3 +/- 21.5 | 237.85 +/- 65.1 | 36.9 +/- 26.5 |
| A204 | 41.8 +/- 5.1 | 218.7 +/- 19.6 | 36.3 +/- 5.5 |
| A204 DasR | >10000 | >10000 | 5010.7+/- 236.7 |
| A204 PazR | >10000 | >10000 | >10000 |
| A204 SunR | >10000 | >10000 | >10000 |

**Table S2. Single and combination drug treatment IC50 concentrations in Pazopanib resistant A204 cell line.**
*Related to Figure 3.*

| Drug treatment | IC50 (nM) |
|---|---|
| Pazopanib | >10000 |
| BGJ398 | 247.4 +/- 29.2 |
| BGJ398 + Pazopanib | 690.1 +/- 133.1 |
| Ponatinib | 271.5 +/- 167.8 |

**Table S3. Variance stabilised RNASeq data of FGFR1 and PDGFRA.**
*Related to Figure 4.*
(Excel File)

**Supplemental Experimental Procedures**

**Cell culture and derivation of acquired resistant sublines**

Cells were cultured in DMEM (A204, G402, Saos2, U2OS, HT1080, SW684, SW872, SW982, Hs729T, RUCH-3, T9195, BT12, CHLA266 and AN3CA), RPMI (G401, RMS-YM and SJSA-1) or McCoy5A (MES-SA) media supplemented with 10% FBS/2mM glutamine/100units/ml penicillin/100mg/ml streptomycin in 95% air/5% $CO_2$ atmosphere at 37°C. For SILAC experiments, A204 cells and resistant sublines were cultured in SILAC DMEM media (Thermo Fisher Scientific) supplemented with light lysine and arginine (R0K0) (Sigma) and heavy lysine and arginine (R10K8) (Goss Scientific) respectively.

Dasatinib, Pazopanib and Sunitinib (LC laboratories) were used to induce resistance in the A204 cells. Cells were grown initially in DMEM media containing drug concentration of 500nM. The drug was incremented when the cells had proliferated to near confluency alongside minimal visible cell death. Drug concentration was incremented from 2µM, 3µM and 5µM in a stepwise manner over 6 weeks. A final drug concentration of 5µM was maintained in resistant cells. Media and drug were replenished twice weekly.

**Molecular biology and lentiviral infection**

The pCDH-EF1-PURO-SMARCB1 plasmid was produced by PCR amplifying the whole SMARCB1 coding sequence from pCDNA 3.1-SMARCB1 (a gift from Frederique Quignon, Institute Curie). Restriction sites for XbaI and BamH1 were added to the Forward and Reverse primers respectively. The PCR product was digested and directionally ligated into the multiple cloning site of pCDH-EF1-Puro (Systems Biosciences).

PCDH-CMV-MCS-EF1-SMARCB1 Puro plasmid (System Bioscences) was transiently transfected into HEK293T cells using Calcium Phosphate Transfection method (CalPhos Transfection Kits, Clontech) according to manufacturer's instructions. Lentiviral infection of rhabdoid cells was carried out aiming to transduce about 60%-80% of the total amount of cells in each experiment, using an MOI of 10. To select for infected cells, Puromycin (Invitrogen) was added to the media to a final concentration of 1µg/mL for 72 hours prior to cell lysis.

**Immunoblotting, immunoprecipitation and immunofluorescence**

For immunoblotting, cells were lysed in RIPA lysis buffer supplemented with protease and phosphatase inhibitors (Thermo Pierce) at $4^oC$. Lysates were loaded onto SDS-PAGE gels followed by blotting onto PVDF membranes as described (Iwai et al., 2013). Blots were probed with primary antibodies followed by corresponding horseradish peroxidase-conjugated secondary antibodies. Primary antibodies include anti-PDGFRα #3174, CST; anti-pAKT (S473) #4058, CST; anti-AKT #4691, CST; anti-pERK-T202/Y204 #4370, CST; anti-ERK #9102, CST; anti-FGFR1 #76464, abcam; anti-BAF47 (SMARCB1) #61211, BD; anti-EGFR#2232, CST; anti-TFR #13-6890, ThermoFisherScientific; anti-pY1000 #8954, CST and anti-α-Tubulin #T5168, Sigma. Secondary antibodies include Polyclonal Goat Anti-Rabbit HRP #P0448, Dako and Anti-Mouse HRP #G32-62G-1000, Signalchem. Immunoreactive bands were visualized by chemiluminescence (Amersham) and the blots were exposed to x-ray XAR film (Kodak).

For immunoprecipitation, cells were lysed in RIPA lysis buffer (containing 1% Triton) supplemented with protease and phosphatase inhibitors (Thermo Pierce) at $4^oC$. After microcentrifugation at 2,000 rpm for 10 min, 200 µg of lysate was diluted in 200ul lysis buffer. Primary antibody (anti-PDGFR#3174, CST) was added at 1mg/ml and incubated with rotation overnight at 4 °C. Protein G plus agarose beads were added and incubated for three hours at 4 °C to collect immune complexes, washed five times with lysis buffer and eluted in sample buffer. Proteins were resolved by SDS–PAGE, transferred to PVDF membrane and immunoblotting was performed as described above.

For immunofluorescence experiments, cells were fixed with 4% formaldehyde for 15 min, permeabilised with 0.2% Triton-X 100/PBS for 10 min and then blocked with IF buffer (3% BSA, 0.05% Tween 20 in PBS) for 1h. Specimens were incubated overnight with primary antibodies (anti-PDGFR #3174, CST; anti-FGFR1 # PA5-18344, Thermo Fisher Scientific) at 4°C rinsed three times with IF buffer and then incubated with secondary antibodies (anti-rabbit Alexa488 and anti-goat Alexa555, Thermo Fisher Scientific). DNA was visualised by DAPI staining. Images were captured using a Zeiss 710 Confocal Microscope.

**Cell viability and apoptosis assays**

For Annexin V staining, 3000 cells/well were seeded into 96-well CellCarrier plates (Perkin Elmer). 24h after seeding, drugs were added and incubated for an additional 48h. Drugs that were used in this study include Dasatinib, Pazopanib, Sunitinib, Ponatinib, AZD4547, BEZ235 and Trametinib from LC laboratories; BGJ398 from Selleck Chemicals. FITC-Annexin V (BD Biosciences) and Hoechst 33342 (Tocris) diluted in 10x annexin binding buffer (0.1M HEPES,

1.4M NaCl, 25mM $CaCl_2$) was added and incubated at 37°C for 15 minutes. Plates were imaged using an Operetta high-content imager (Perkin Elmer). Images were analysed using Harmony software (Perkin Elmer), and annexin positivity defined as number of annexin-FITC-positive cells relative to total number of Hoechst-positive nuclei. The interaction between drugs was analysed by the Chou and Talalay median effect principle as described (Todd et al., 2014).

siRNA transfections were performed as follows, 2000 cells/well were reverse transfected in 96-well plates with SMARTpool siRNAs (Dharmacon) using Lullaby reagent (Oz Biosciences). Where indicated, cells were treated with vehicle or drug 24h post transfection. Apoptosis and cell viability were measured using Caspase 3/7 Glo and Cell Titre Glo (Promega), respectively, 72-96h post transfection according to manufacturer's instructions and normalised to cells transfected with a non-targeting siRNA pool.

**aCGH, gene expression and phosphoproteomic analysis**

For aCGH analysis, genomic DNA was extracted as previously described (Marchio et al., 2008; Natrajan et al., 2009). The aCGH platform was constructed in-house and comprises ~32,000 BAC clones tiled across the genome. This platform has been shown to be as robust as, and to have comparable resolution with, high-density oligonucleotide arrays (Coe et al., 2007; Gunnarsson et al., 2008). aCGH data were pre-processed and analyzed using the Base.R script in R version 2.14.0, as previously described (Natrajan et al., 2014). Genomic DNA from each sample was hybridized against a pool of normal female DNA derived from peripheral blood. Raw $Log_2$ ratios of intensity between samples and pooled female genomic DNA were read without background subtraction and normalized in the LIMMA package in R using PrinTipLoess. Outliers were removed based upon their deviation from neighboring genomic probes, using an estimation of the genome-wide median absolute deviation of all probes. $Log_2$ ratios were rescaled using the genome wide median absolute deviation in each sample and then smoothed using circular binary segmentation (cbs) in the DNACopy package as described (Natrajan et al., 2009). After filtering polymorphic BACs and BACs mapping to chromosome Y, a final dataset of 31,157 clones with unambiguous mapping information according to build hg19 of the human genome (http://www.ensembl.org). A categorical analysis was applied to the BACs after classifying them as representing amplification (>0.45), gain (>0.08 and ≤0.45), loss (<−0.08) or no change, according to their cbs-smoothed log2 ratio values (Marchio et al., 2008; Natrajan et al., 2009). Threshold values were determined and validated as previously described (Natrajan et al., 2009).

The Illumina Bead Chip (HumanHG-12 v4) data were pre-processed, log2-transformed, and quantile normalized using the *beadarray* package in Bioconductor (Dunning et al., 2007). We performed hierarchical clustering of the data using the MATLAB bioinformatics toolbox with Euclidean distance metric and average linkage to generate the hierarchical tree. Data rows (genes) were normalized so that the mean was 0 and the standard deviation was 1. Gene expression data has been deposited into the GEO repository, accession number GSE78864. Gene Set Enrichment Analysis (GSEA) software was used to identify the 50 genes with the strongest positive correlations and 50 genes with the strongest negative correlations to the phenotype (i.e. drug resistance). We ranked genes using the Signal2Noise (default) option in GSEA. Signal2Noise uses the difference of means scaled by the standard deviation for ranking defined as (mean1-mean2)/(st.dev1 + st.dev2). Statistical significance of gene correlations to the drug resistance phenotype (top 100 genes) were determined using the False Discovery Rate (FDR) method which corrects for multiple comparisons.

Phosphotyrosine proteomic analysis was performed as previously described (Iwai et al., 2013) with the following modifications: SILAC labelled cells (biological triplicates) were lysed in 8M urea and equal amounts of heavy (DasR or PasR cells) and light (parental cells) lysates were mixed prior to reduction, alkylation and trypsin digestion. Peptides were desalted on a C18 cartridge, eluted with 25% acetonitrile and lyophilised to dryness. A two-step enrichment of phosphotyrosine peptides was performed; immunoprecipitation (IP) using a combination of pTyr100, pTyr1000 (Cell Signalling) and 4G10 (Millipore) followed by immobilized metal affinity chromatography (IMAC) on $FeCl_3$ charged NTA beads as previously described (Iwai et al 2013). Eluted peptides were then subjected to reverse-phase liquid chromatography separation (Iwai et al 2013) followed by electrospray ionization and MS/MS on a Triple-TOF 5600+ mass spectrometer (ABSciex) operated in a data-dependent acquisition mode with top 25 most intense peaks (two to five positive charges) automatically acquired with previously selected peaks excluded for 30s. Detailed MS data related to Figure 2C is available upon request.

The data were processed with MaxQuant (Cox and Mann, 2008)(version 1.5.2.8) and the peptides were identified (maximal mass error = 0.006 Da and 40 ppm for precursor and product ions, respectively) from the MS/MS spectra searched against human referenced proteome (UniProt, June 2015) using Andromeda (Cox et al., 2011) search engine. The following peptide bond cleavages: arginine or lysine followed by any amino acid (a general setting referred to as Trypsin/P) and up to two missed cleavages were allowed. SILAC based experiments in MaxQuant were performed using the built-in quantification algorithm (Cox and Mann 2008) with minimal ratio count = 2 and enabled 'Re-quantify' feature. Cysteine carbamidomethylation was selected as a fixed modification whereas methionine oxidation, acetylation of protein N-terminus and phospho (STY) as variable modifications. The false discovery rate was set to 0.01

for peptides, proteins and sites. Other parameters were used as pre-set in the software. "Unique and razor peptides" mode was selected to allow identification and quantification of proteins in groups.

Data were further analysed using Microsoft Office Excel 2007 and Perseus (version 1.5.0.9). The data were filtered to remove potential contaminants and IDs originating from reverse decoy sequences. The log2 values of the heavy/light (H/L) ratios were then determined. An arbitrary value of +10 or -10 was manually imputed when only H or L intensity, respectively, was detected and thus the H/L ratio could not have been automatically assigned by MaxQuant. The data were then normalized to the average H/L ratio of the total proteome (IP supernatant) and filtered to include only high confidence phosphosite IDs (localization probability and score difference ≥ 90% and 10, respectively). For generation of the heat map (Figure 2C), normalized H/L ratios of respective triplicates were averaged and reversed (L/H) to visualize the log2 fold changes in phosphorylation between parental (L) and resistant (H) cells.

**RNA Seq and immunohistochemistry analysis of patient specimens**

RNA was extracted using a Qiagen all-prep kit from 23 fresh frozen tumour tissue samples taken from paediatric patients with a confirmed diagnosis of SMARCB1 negative MRT. A paired end cDNA sequencing library was created using Illumina's Tru-seqv2 RNA library preparation kit according to manufacturer's instructions. Libraries were sequenced on an Illumina Hi-Seq2500 to give ~90M paired end reads. Reads were QC checked (FastQC) and aligned using RNA-STAR (Dobin et al., 2013), and gene counts generated using Gencodev19 Transcriptome library and HTSeq-count (Anders et al., 2015). Library normalisation, dispersion estimations and variance stabilising transformations were generated using R Bioconductor package DESeq2 (Love et al., 2014) as were adjusted P-values and moderated log fold change values calculated by one-sided t-test to indicate significant differences in expression between RT samples and the normal tissue collection. RNA-seq data from normal tissues were taken from Illumina Bodymap (ArrayExpress E-MTAB-513, E-MTAB-2836). Variance stabilised RNASeq data is provided in Table S3.

The fully automated Ventana BenchMark XT IHC system and standard detection reagents ultraVIEW™ Universal DAB Detection kit (Ventana Medical Systems; Cat No: 760-500) were used to demonstrate SMARCB1 (BAF47 BD Transduction Labs Cat No: 612111) in 4 micrometre tissue sections at a dilution of 1/100. This incorporated antigen retrieval with Ventana ultra cell conditioning 1 (64 minutes) and a haematoxylin counterstain (Ventana Medical Systems: Cat No: 950-224). PDGFRα and FGFR1 were both demonstrated using the Dako Autostainer Link48 platform. Briefly, slides were antigen retrieved using pH6 citrate, either via microwave (PDGFRα) or MenaPath Access Retrieval Unit (FGFR1) before being placed onto the autostainer where they were incubated in primary antibody, diluted 1/250 for PDGFRα (3714 Cell Signalling) and 1/50 for FGFR1 (ab76464 Abcam) and detected using Dako EnVision FLEX reagents (Dako K8002).

**Supplemental References**


Anders, S., Pyl, P.T., and Huber, W. (2015). HTSeq--a Python framework to work with high-throughput sequencing data. Bioinformatics *31*, 166-169.

Coe, B.P., Ylstra, B., Carvalho, B., Meijer, G.A., Macaulay, C., and Lam, W.L. (2007). Resolving the resolution of array CGH. Genomics *89*, 647-653.

Cox, J., and Mann, M. (2008). MaxQuant enables high peptide identification rates, individualized p.p.b.-range mass accuracies and proteome-wide protein quantification. Nat Biotechnol *26*, 1367-1372.

Cox, J., Neuhauser, N., Michalski, A., Scheltema, R.A., Olsen, J.V., and Mann, M. (2011). Andromeda: a peptide search engine integrated into the MaxQuant environment. J Proteome Res *10*, 1794-1805.

Dobin, A., Davis, C.A., Schlesinger, F., Drenkow, J., Zaleski, C., Jha, S., Batut, P., Chaisson, M., and Gingeras, T.R. (2013). STAR: ultrafast universal RNA-seq aligner. Bioinformatics *29*, 15-21.

Dunning, M.J., Smith, M.L., Ritchie, M.E., and Tavare, S. (2007). beadarray: R classes and methods for Illumina bead-based data. Bioinformatics *23*, 2183-2184.

Gunnarsson, R., Staaf, J., Jansson, M., Ottesen, A.M., Goransson, H., Liljedahl, U., Ralfkiaer, U., Mansouri, M., Buhl, A.M., Smedby, K.E., *et al.* (2008). Screening for copy-number alterations and loss of heterozygosity in chronic lymphocytic leukemia--a comparative study of four differently designed, high resolution microarray platforms. Genes Chromosomes Cancer *47*, 697-711.

Iwai, L.K., Payne, L.S., Luczynski, M.T., Chang, F., Xu, H., Clinton, R.W., Paul, A., Esposito, E.A., Gridley, S., Leitinger, B., *et al.* (2013). Phosphoproteomics of collagen receptor networks reveals SHP-2 phosphorylation downstream of wild-type DDR2 and its lung cancer mutants. Biochem J *454*, 501-513.

Love, M.I., Huber, W., and Anders, S. (2014). Moderated estimation of fold change and dispersion for RNA-seq data with DESeq2. Genome Biol *15*, 550.

Marchio, C., Iravani, M., Natrajan, R., Lambros, M.B., Savage, K., Tamber, N., Fenwick, K., Mackay, A., Senetta, R., Di Palma, S., *et al.* (2008). Genomic and immunophenotypical characterization of pure micropapillary carcinomas of the breast. J Pathol *215*, 398-410.

Natrajan, R., Lambros, M.B., Rodriguez-Pinilla, S.M., Moreno-Bueno, G., Tan, D.S., Marchio, C., Vatcheva, R., Rayter, S., Mahler-Araujo, B., Fulford, L.G., *et al.* (2009). Tiling path genomic profiling of grade 3 invasive ductal breast cancers. Clin Cancer Res *15*, 2711-2722.

Natrajan, R., Wilkerson, P.M., Marchio, C., Piscuoglio, S., Ng, C.K., Wai, P., Lambros, M.B., Samartzis, E.P., Dedes, K.J., Frankum, J., *et al.* (2014). Characterization of the genomic features and expressed fusion genes in micropapillary carcinomas of the breast. J Pathol *232*, 553-565.

Todd, J.R., Scurr, L.L., Becker, T.M., Kefford, R.F., and Rizos, H. (2014). The MAPK pathway functions as a redundant survival signal that reinforces the PI3K cascade in c-Kit mutant melanoma. Oncogene *33*, 236-245.
