# Supplementary material for: Dual Targeting of PDGFRα and FGFR1 Displays Synergistic Efficacy in Malignant Rhabdoid Tumors
Source: Cell Rep. 2016 Oct 25;17(5):1265–75. doi: 10.1016/j.celrep.2016.10.005 (PMC5098123; doi:10.1016/j.celrep.2016.10.005)
Supplement: Document S2. Article plus Supplemental Information [file mmc3.pdf]

Cell Reports

Report

Dual Targeting of PDGFRα and FGFR1 Displays Synergistic Efficacy in Malignant Rhabdoid Tumors

Graphical Abstract

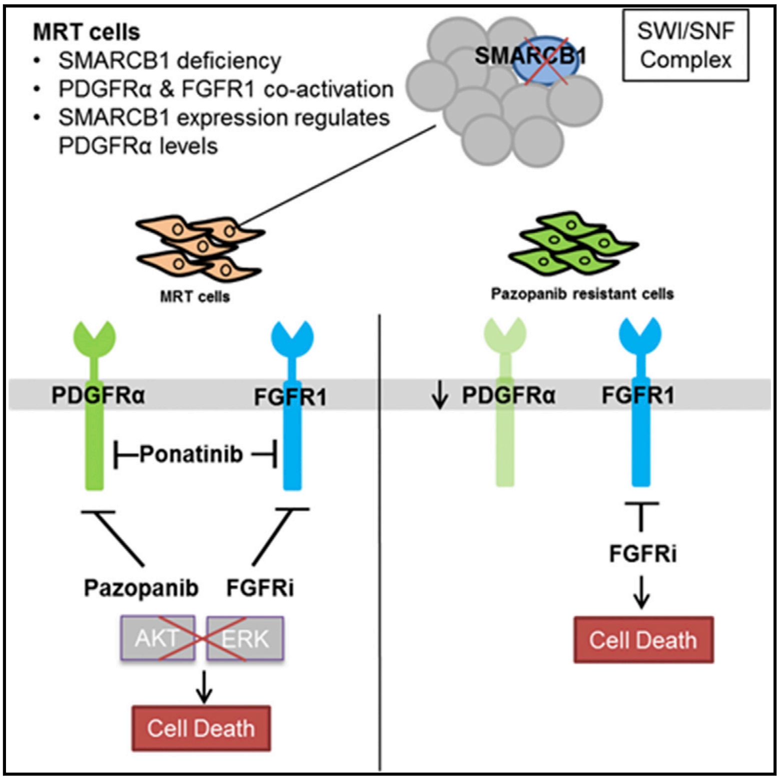

Authors

Jocelyn P. Wong, Jason R. Todd, Martina A. Finetti, ..., Rachael C. Natrajan, Daniel Williamson, Paul H. Huang

Correspondence

paul.huang@icr.ac.uk

In Brief

Malignant rhabdoid tumors (MRTs) are pediatric cancers characterized by a deficiency in the SWI/SNF subunit SMARCB1. Wong et al. show that MRTs display coactivation of PDGFRα and FGFR1 and that dual blockade of these receptors induces apoptosis. These findings present therapeutic opportunities to exploit tyrosine kinase dependencies in cancers with SWI/SNF deficiencies.

Highlights

- Malignant rhabdoid tumors display coactivation of PDGFRα and FGFR1
- Dual inhibition of PDGFRα and FGFR1 leads to synergistic apoptosis
- FGFR1 inhibition overcomes acquired resistance to pazopanib treatment
- PDGFRα and FGFR1 are expressed in rhabdoid tumor patient specimens

Accession Numbers

GSE78864
GSE87208

Wong et al., 2016, Cell Reports *17*, 1265–1275
October 25, 2016 © 2016 The Author(s).
http://dx.doi.org/10.1016/j.celrep.2016.10.005

CellPress

Cell Reports
Report

OPEN ACCESS
CellPress

Dual Targeting of PDGFRα and FGFR1 Displays Synergistic Efficacy in Malignant Rhabdoid Tumors

Jocelyn P. Wong,[1,6] Jason R. Todd,[1,6] Martina A. Finetti,[2] Frank McCarthy,[1] Malgorzata Broncel,[1] Simon Vyse,[1] Maciej T. Luczynski,[1] Stephen Crosier,[2] Karen A. Ryall,[3] Kate Holmes,[1] Leo S. Payne,[1] Frances Daley,[4] Patty Wai,[4] Andrew Jenks,[5] Barbara Tanos,[5] Aik-Choon Tan,[3] Rachael C. Natrajan,[4] Daniel Williamson,[2] and Paul H. Huang[1,7,*]
[1]Division of Cancer Biology, The Institute of Cancer Research, London SW3 6JB, UK
[2]Northern Institute for Cancer Research, Newcastle University, Newcastle upon Tyne NE1 4LP, UK
[3]Translational Bioinformatics and Cancer Systems Biology Laboratory, Division of Medical Oncology, Department of Medicine, University of Colorado Anschutz Medical Campus, Aurora, CO 80045, USA
[4]The Breast Cancer Now Toby Robins Research Centre, Division of Breast Cancer Research, The Institute of Cancer Research, London SW3 6JB, UK
[5]Division of Cancer Therapeutics, The Institute of Cancer Research, London SW3 6JB, UK
[6]Co-first author
[7]Lead Contact
*Correspondence: paul.huang@icr.ac.uk
http://dx.doi.org/10.1016/j.celrep.2016.10.005

SUMMARY

Subunits of the SWI/SNF chromatin remodeling complex are mutated in a significant proportion of human cancers. Malignant rhabdoid tumors (MRTs) are lethal pediatric cancers characterized by a deficiency in the SWI/SNF subunit SMARCB1. Here, we employ an integrated molecular profiling and chemical biology approach to demonstrate that the receptor tyrosine kinases (RTKs) PDGFRα and FGFR1 are coactivated in MRT cells and that dual blockade of these receptors has synergistic efficacy. Inhibitor combinations targeting both receptors and the dual inhibitor ponatinib suppress the AKT and ERK1/2 pathways leading to apoptosis. MRT cells that have acquired resistance to the PDGFRα inhibitor pazopanib are susceptible to FGFR inhibitors. We show that PDGFRα levels are regulated by SMARCB1 expression, and assessment of clinical specimens documents the expression of both PDGFRα and FGFR1 in rhabdoid tumor patients. Our findings support a therapeutic approach in cancers with SWI/SNF deficiencies by exploiting RTK coactivation dependencies.

INTRODUCTION

Inactivating mutations in genes encoding components of the SWI/SNF chromatin remodeling complex are found in ~20% of cancers (Kadoch et al., 2013). Treatment of this class of tumors is challenging and there are currently no targeted therapies approved for clinical use. The prototypical example of this class is the malignant rhabdoid tumors (MRTs), which are rare pediatric cancers of the kidney and soft tissues. MRTs are characterized by the biallelic inactivation of the *SMARCB1* (*INI1/SNF5/BAF47*) gene, which encodes a core component of the SWI/SNF complex and is a tumor suppressor (Kim and Roberts, 2014).

In addition to the MRTs, atypical teratoid/rhabdoid tumors (AT/RTs) are rhabdoid tumors of the CNS that are similarly characterized by the loss of SMARCB1 (Frühwald et al., 2016). MRTs are highly aggressive, and, despite intensive multimodal therapy, prognosis remains dismal with many children not surviving beyond 12 months (Madigan et al., 2007). SMARCB1 mutation is the sole driver of disease, and MRTs and AT/RTs lack additional gene amplifications or deletions and demonstrate low rates of mutations (Chun et al., 2016; Johann et al., 2016; Lee et al., 2012). The mechanisms by which SMARCB1 loss contributes to tumor progression are not fully understood, and analyses of genes regulated by SMARCB1 have revealed several candidate oncogenes, including components of the cell cycle machinery, sonic hedgehog pathway, and canonical Wnt signaling (Kim and Roberts, 2014). Identifying the fundamental oncogenic drivers resulting from SMARCB1 deficiency remains a significant challenge and a key barrier to developing effective therapies.

Receptor tyrosine kinases (RTKs) are attractive targets for cancer therapy with several tyrosine kinase inhibitors (TKIs) clinically approved for a range of tumor types (Lemmon and Schlessinger, 2010). We and others have shown that cancer cells rely on the activation of multiple RTKs to maintain robust oncogenic signaling (Huang et al., 2007). Employing TKI combinations is effective in overcoming compensatory RTK signaling and ultimately killing cancer cells (Xu and Huang, 2010). In this study, we utilize an integrated molecular profiling and chemical biology approach to show that MRT cells display coactivation of PDGFRα and FGFR1 and that therapeutic inhibition of both RTKs leads to synergistic cytotoxicity. Our findings demonstrate that exploiting RTK coactivation dependencies may be beneficial in the treatment of cancers with SWI/SNF deficiencies.

© 2016 The Author(s).
This is an open access article under the CC BY license (http://creativecommons.org/licenses/by/4.0/).

RESULTS

MRT Cell Lines Are Selectively Responsive to Dasatinib, Pazopanib, and Sunitinib

The TKIs dasatinib, pazopanib, and sunitinib are either approved or currently being evaluated for soft tissue malignancies, such as sarcomas and MRTs. To identify subtypes that may be selectively responsive to these TKIs, a panel of 14 sarcoma and MRT lines were subjected to dose-response assessment. Only the MRT cell lines A204 and G402 were found to be sensitive to all three TKIs (Figure 1A; Table S1).

Analysis of Acquired Resistance Identifies PDGFRα as an Oncogenic Driver in MRT Cells

Durable responses to TKIs are rare and most patients develop acquired drug resistance (Kasper et al., 2014). To discover potential resistance mechanisms, we modeled acquired resistance in vitro by subjecting the A204 cells to long-term escalating dose treatment with each of the three TKIs. Cell viability analysis confirmed that these sublines have acquired resistance and were cross-resistant to each other (Figure 1B; Table S1), suggesting a common mechanism of action.

To identify candidate kinases that confer TKI sensitivity, we assessed the target selectivity overlap among the three inhibitors based on published screens of TKI selectivity (Anastassiadis et al., 2011; Davis et al., 2011). Pazopanib, dasatinib, and sunitinib share three common RTK targets, KIT, CSF1R, and PDGFRα (Figure 1C), of which only PDGFRα is activated in the A204 cells as shown by a previous phosphoproteomic screen (Bai et al., 2012). Immunoblotting revealed a reduction in PDGFRα expression in the acquired resistant sublines (Figure 1D), indicating that a loss in PDGFRα pathway dependency is a potential mechanism of drug resistance.

Treatment of the parental A204 cells with the three TKIs led to a decrease in PDGFRα phosphorylation (Figure 1E). Furthermore, small interfering RNA (siRNA) depletion of PDGFRα was able to phenocopy the TKI effects and decrease MRT cell viability (Figures 1F and 1G). Immunoblot analysis of downstream signaling components AKT and ERK1/2, which control cell proliferation and survival, showed that the TKIs abolished AKT phosphorylation but had no effect on ERK1/2 phosphorylation in the parental cells (Figure 1H). Upon ectopic expression of SMARCB1 in the MRT cells, PDGFRα levels were decreased compared to control (Figure 1I), demonstrating that SMARCB1 regulates PDGFRα expression. Collectively, our findings show that PDFGRα is a driver in MRT cells that is regulated by SMARCB1 and can be effectively inhibited using pazopanib, dasatinib, and sunitinib.

Molecular Profiling of A204 Parental and Resistant Cells

To identify additional candidate drivers in MRTs, we undertook a molecular profiling strategy comprising microarray-based comparative genomic hybridization (aCGH), gene expression analysis, and phosphoproteomics, using the A204 parental and three resistant sublines as a model. The aCGH was performed to assess chromosomal gains or losses associated with acquired resistance. The A204 cells have a simple genome with no detectable chromosomal alterations other than a focal deletion of *SMARCB1* at 22q11.23 (Figures 2A and S1A), which is maintained in the resistant sublines. Of the resistant cells, only the dasatinib-resistant (DasR) subline harbored additional gains on chromosome 17q21.32-q25.3 and losses of the whole arm of 13q (Figure 2A). Since this genomic profile was specific to DasR, it is unlikely that any targets identified in these chromosomal regions would be common to all three TKIs and, thus, were not pursued further. Gene expression analysis of the four cell lines in the presence of TKI showed that the resistant sublines clustered together with the untreated parental cells (Figure S1B), and it confirmed that *PDGFRA* was among the most highly downregulated genes in the resistant cells (Figures 2B and S1C).

Phosphoproteomics was used to compare the signaling profiles of DasR and pazopanib-resistant (PazR) sublines versus parental cells. Sunitinib-resistant (SunR) cells were not analyzed because their low proliferation rate prevented sufficient cells from being harvested. We show that parental cells displayed high levels of phosphorylated PDGFRα at multiple sites (Y613, Y742, Y762, Y768, and Y849) (Figure 2C). Interestingly, FGFR1 phosphorylation in the kinase insert domain (Y583 and Y585) also was found to be elevated in the parental cells. Additionally, FGFR1 was phosphorylated in its activation loop (Y653 and Y654) at similar levels in both parental and resistant cells. These data confirm that PDGFRα is the only common kinase target of pazopanib, dasatinib, and sunitinib that is activated in these cells (Figure 1C), and they demonstrate that both PDGFRα and FGFR1 are coactivated with multiple phosphosites observed in each receptor.

Dual Targeting of PDGFRα and FGFR1 Enhances Apoptosis

Since FGFR1 phosphorylation was uncovered in our phosphoproteomic analysis, coupled with a previous report that FGFR RTKs are therapeutic targets in MRTs (Wöhrle et al., 2013), we reasoned that a combination of PDGFRα and FGFR inhibitors may have enhanced efficacy. We first assessed the effects of two selective FGFR TKIs NVP-BGJ398 and AZD4547 on the viability of A204 and G402 cells (Tan et al., 2014). AZD4547 was ineffective in both cell lines while BGJ398 only reduced viability in the A204 cells (Figure 3A). As a positive control, AN3CA cells that harbor an FGFR2 mutation and are sensitive to FGFR TKIs were used (Tan et al., 2014). Depletion of FGFR1 using siRNA also showed a minor decrease in the viability of the MRT cells (Figures 3B and 3C).

We evaluated the effects of BGJ398 and AZD4547 in combination with PDGFRα TKIs on cell viability and apoptosis. This combination showed a small decrease in A204 and G402 viability compared to single inhibitor treatment (Figure S2A), reflecting the strong cytostatic consequence of PDGFRα TKI monotherapy (Figure 1A). Assessment of caspase 3/7 activity found that PDGFRα or FGFR TKI treatment alone led to low levels of apoptosis despite high drug concentrations of up to 1 μM (Figures 3D and S2B). Dual PDGFRα and FGFR inhibition showed significantly increased apoptosis (>6-fold relative to vehicle control). This enhanced apoptosis was recapitulated with a combination of siRNA depletion of PDGFRα and BGJ398 or AZD4547 treatment (Figure S2C). To assess if the combination confers synergistic cytotoxicity in the A204 cells, we employed an

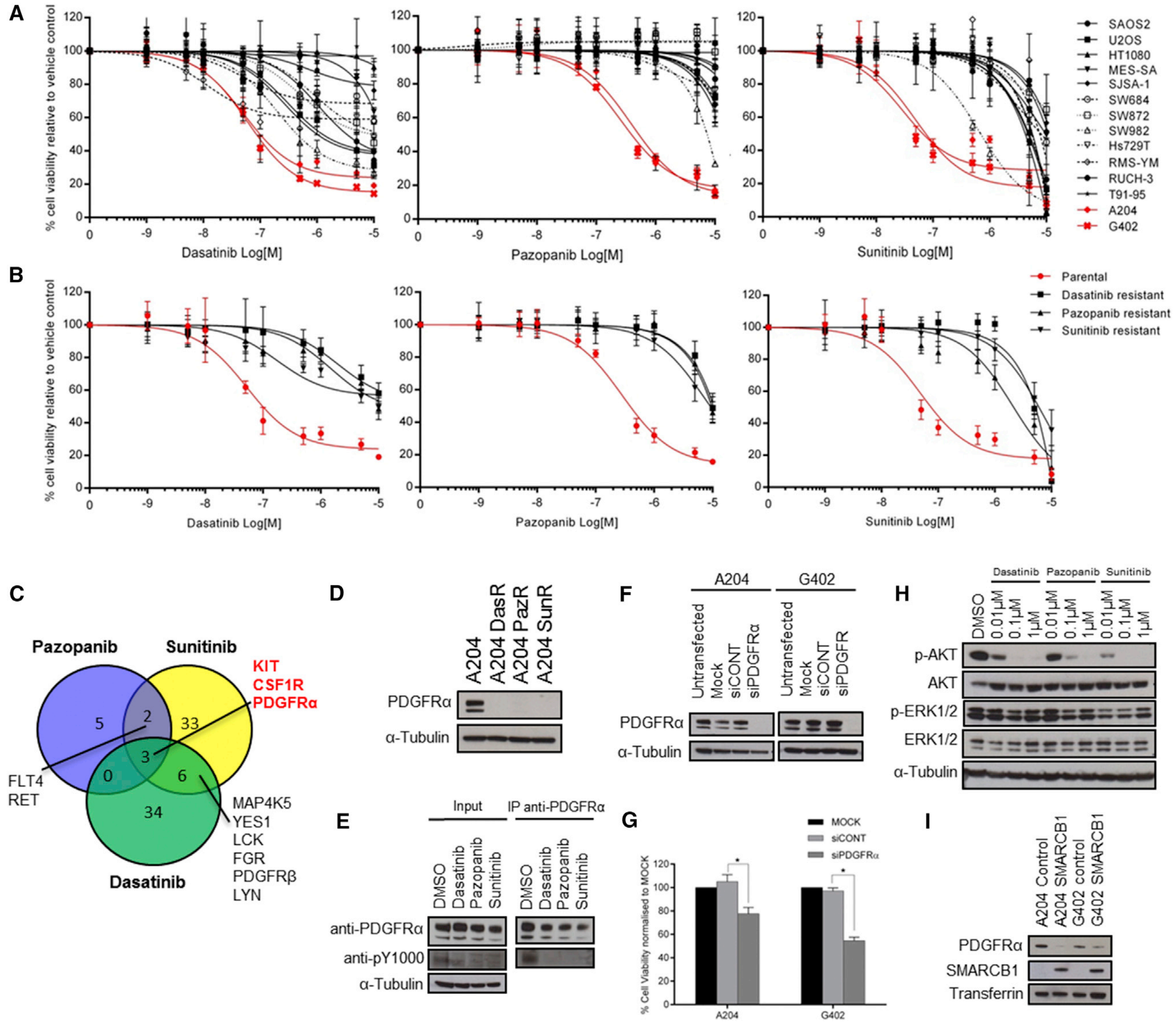

Figure 1. MRT Cell Lines Are Sensitive to PDGFRα Inhibitors

(A) Dose-response curves of dasatinib-, pazopanib-, and sunitinib-resistant (black) and -sensitive (red) cell lines. A panel of 14 cell lines was treated with a range of drug concentrations to determine IC_{50} values (Table S1). Cell viability is normalized to DMSO control (n = 2 or 3).

(B) Dose-response curves of TKI-resistant sublines (black) and parental A204 cells (red). IC_{50} values are detailed in Table S1. Cell viability is normalized to DMSO control (n = 3).

(C) Target selectivity overlap plot of dasatinib, pazopanib, and sunitinib shows that KIT, CSF1R, and PDGFRα are common targets.

(D) Immunoblot of PDGFRα expression in parental A204 and resistant sublines is shown. DasR, dasatinib resistant; PazR, pazopanib resistant; SunR, sunitinib resistant.

(E) Immunoprecipitation of PDGFRα followed by immunoblotting with phosphotyrosine-specific antibody (PY1000) shows a decrease in receptor phosphorylation with 1 μM TKI for 1 hr.

(F) Immunoblot of PDGFRα expression in the MRT cells under mock, non-targeting control siCONT and siPDGFRα pool transfection conditions is shown.

(G) Bar plots showing cell viability of MRT cells upon siRNA silencing of PDGFRα. Cell viability data are normalized to mock transfection (n = 3). Statistical analysis of siPDGFRα versus siCONT was performed by paired Student's t test (*$p < 0.05$).

(H) Immunoblot of AKT and ERK1/2 phosphorylation levels in A204 cells treated with TKIs at the indicated doses for 3 hr is shown.

(I) Immunoblot of PDGFRα shows downregulation of receptor levels upon ectopic SMARCB1 expression.

For (A), (B), and (G), all values are mean ± SD.

automated imaging assay to visualize annexin V-positive cells. While the individual TKIs only resulted in <5% apoptotic cells (Figure S2D), the combination of BGJ398 with either pazopanib or dasatinib led to a synergistic increase (combination index < 1) in the proportion of apoptotic cells to ~30%–50% across all drug doses tested (Figures 3E and S2D).

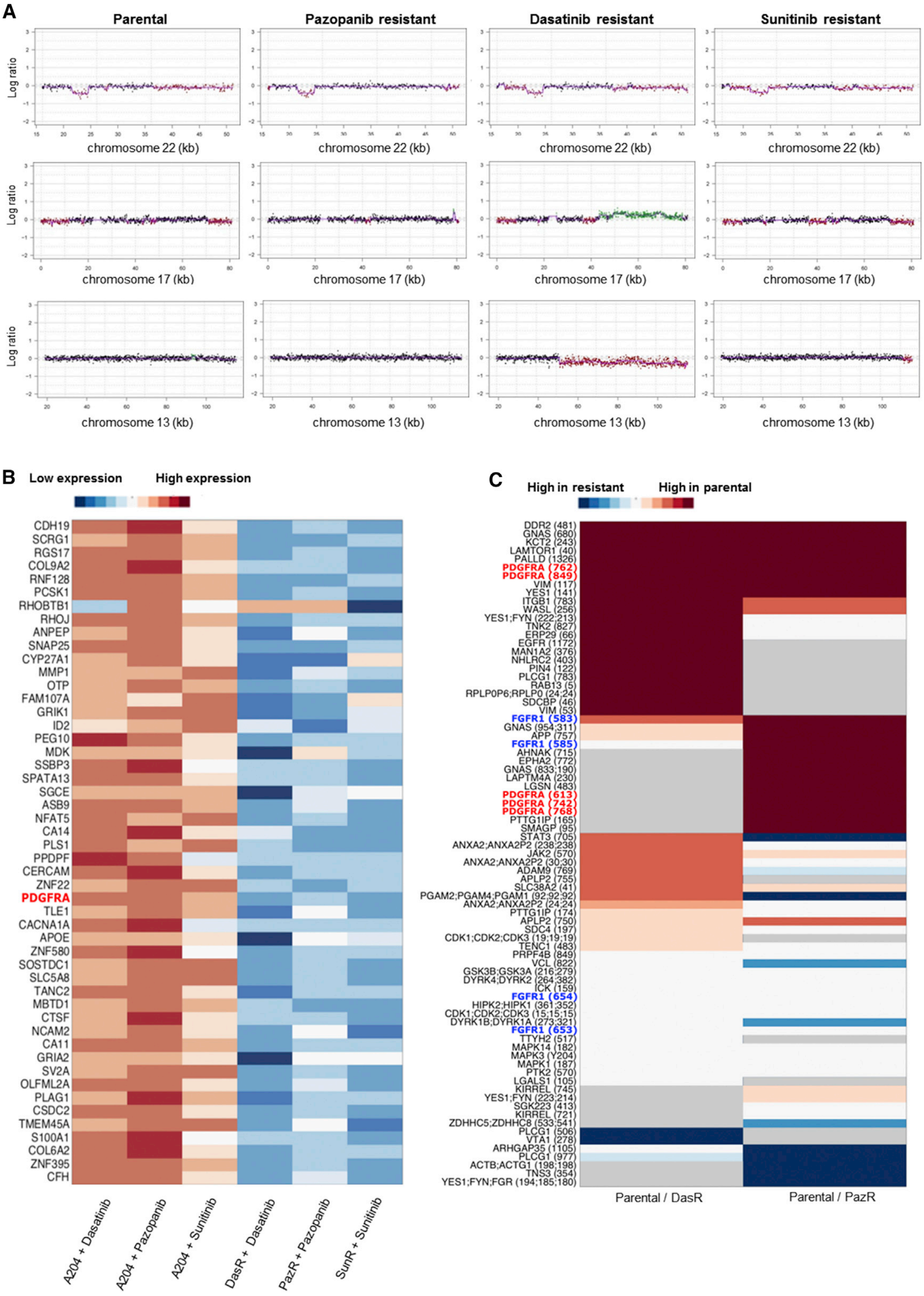

Figure 2. Molecular Profiling of A204 Cells

(A) aCGH plots of A204 parental and resistant cells. Selected profiles of chromosome 22 illustrate focal deletion of SMARCB1 in 22q11.23. DasR harbors chromosome 17 and 13 alterations, illustrating gains (green) and losses (red), respectively. Full genomic profiles are presented in Figure S1A.

(legend continued on next page)

To establish if a dual inhibitor of both receptors is capable of inducing apoptosis as a single agent, the effects of ponatinib, a potent inhibitor of FGFR1 and PDGFRα (Gozgit et al., 2011), were investigated. While previous reports claim that pazopanib and sunitinib are FGFR1 inhibitors, the K_D of these compounds for FGFR1 are 128-fold and 67-fold higher, respectively, compared to ponatinib (Tucker et al., 2014). Assessing the dose-response effects of ponatinib in the panel of 17 cell lines, consisting of five SMARCB1-deficient cell lines (A204, G402, G401, BT12, and CHLA226) and 12 wild-type sarcoma cell lines, confirmed that cells with SMARCB1 deficiency were sensitive to this TKI (Figure 3F). Treatment with ponatinib resulted in enhanced apoptosis in the A204 and G402 MRT cells, at levels similar to combined PDGFRα and FGFR TKI treatment (Figures 3G and S2E).

In contrast to the PDGFRα TKIs, FGFR inhibitor (BGJ398) treatment had no effect on AKT phosphorylation but instead decreased ERK1/2 phosphorylation (Figure 3H). As expected, BGJ398 had no effects on PDGFRα phosphorylation (Figure S2F). Correspondingly, combined treatment with PDGFRα and FGFR TKIs or ponatinib resulted in the suppression of both ERK1/2 and AKT phosphorylation (Figure 3H), consistent with a model where inhibition of both pathways is required for inducing apoptosis in MRT cells. To test this hypothesis, we treated the A204 cells with the PI3K/mTOR inhibitor NVP-BEZ235, the MEK inhibitor trametinib, and a combination of both inhibitors to block the AKT and ERK1/2 pathways, respectively. Immunoblotting confirmed that these pathways were suppressed upon inhibitor treatment (Figure 3I). Similar to PDGFRα inhibitor monotherapy (Figures 1A and S2A), treatment with BEZ235 alone led to a decrease in cell viability (Figure 3J) but had limited effects on apoptosis (Figure 3K). Treatment with trametinib alone was ineffective in reducing cell viability (Figure 3I), which is consistent with FGFR inhibitor monotherapy data (Figure 3A). Combined treatment of BEZ235 and trametinib recapitulates the elevated apoptosis levels (Figure 3K) observed with ponatinib or PDGFRα and FGFR inhibitor combinations (Figures 3D and 3G). Collectively our data provide additional support that MRTs cells require both the AKT and ERK1/2 pathways for cell survival.

FGFR Inhibitors Sensitize MRT Cells that Have Acquired Resistance to Pazopanib

Given that pazopanib is approved for soft tissue malignancies and there is currently no effective means to treat patients whose tumors have progressed on this TKI, we investigated if targeting FGFR1 is capable of sensitizing cells that have acquired pazopanib resistance. The resistant sublines maintain FGFR1 expression (Figure S3A) and activation loop phosphorylation (Figure 2C) at levels similar to the parental cells. Treating PazR cells with BGJ398 led to a reduction in cell viability that was not enhanced by the addition of pazopanib, demonstrating that these cells are no longer addicted to PDGFRα (Figure 3L; Table S2). The degree of sensitization of the PazR cells in response to BGJ398 was similar to the IC_{50} of pazopanib treatment in the parental A204 cells (Table S1). Pazopanib alone had no effect on apoptosis compared to vehicle control, while BGJ398, ponatinib, or the combination of BGJ398 and pazopanib led to a significant increase in the proportion of apoptotic cells (Figure 3M). These data demonstrate that FGFR1 blockade is an effective means of overcoming resistance to pazopanib.

Since the AKT pathway is inhibited by pazopanib via PDGFRα blockade (Figure 3H), we sought to determine if bypass of the requirement for the AKT pathway is a potential mechanism of pazopanib resistance. In the absence of pazopanib, PazR cells maintained a reduced level of AKT phosphorylation (compared to parental A204 cells), which decreased upon treatment with pazopanib, while FGFR1 blockade did not reduce AKT phosphorylation levels (Figure S3B). Treatment of PazR cells with BEZ235 abolished AKT phosphorylation (Figure S3C). Dose-response measurements showed that PazR cells were more resistant to BEZ235 treatment compared with parental A204 cells, with a >3-fold increase in IC_{50} values (Figure S3D). In addition, BEZ235 treatment in PazR cells did not lead to a statistically significant increase in apoptosis levels compared to parental A204 cells (Figure S3E). These data demonstrate that one potential mechanism of pazopanib resistance in the PazR cells is a reduced requirement for the AKT pathway for cell survival.

In some cancers, subpopulations of cancer cells display mutually exclusive RTK amplification events reflecting intratumoral heterogeneity, and clonal selection during therapy leads to acquired resistance (Szerlip et al., 2012). Previous fluorescence in situ hybridization (FISH) analysis of A204 cells found that PDGFRα was not amplified at the genomic level (McDermott et al., 2009). To establish if heterogeneity in RTK expression could be a potential mechanism for drug resistance, immunofluorescence was performed to determine the distribution of PDGFRα and FGFR1. We found that both RTKs were expressed in all cells within the parental A204 population (Figure S3F), and, consistent with the immunoblot data, the three resistant sublines displayed reduced PDGFRα levels and maintained FGFR1 expression. These data confirm that RTK expression is not mutually exclusive in distinct subpopulations, and they suggest that acquired resistance is unlikely the result of clonal selection of a pre-existing PDGFRα-deficient subpopulation but rather the consequence of genetic evolution by PDGFRα loss in drug-tolerant cells during TKI selection (Hata et al., 2016).

PDGFRA and FGFR1 Are Expressed in Rhabdoid Tumor Patients

To verify the clinical relevance of our findings, we evaluated the mRNA levels of *PDGFRA* and *FGFR1* in an RNA sequencing (RNA-seq) dataset of 23 primary rhabdoid tumor (RT) patient

(B) Heatmap of the top 50 downregulated genes in the resistant sublines versus the parental A204 cells treated with TKIs. Full gene expression dataset is presented in Figure S1B.

(C) Heatmap of phosphoproteomic data with log_2 fold change of untreated A204 parental cells versus DasR or PazR in the presence of TKI versus with PDGFRα and FGFR1 phosphorylation sites highlighted in red and blue, respectively. Gray boxes represent phosphosites that were not observed under that specific condition. Data presented are an average of three independent experiments.

OPEN
ACCESS
CellPress

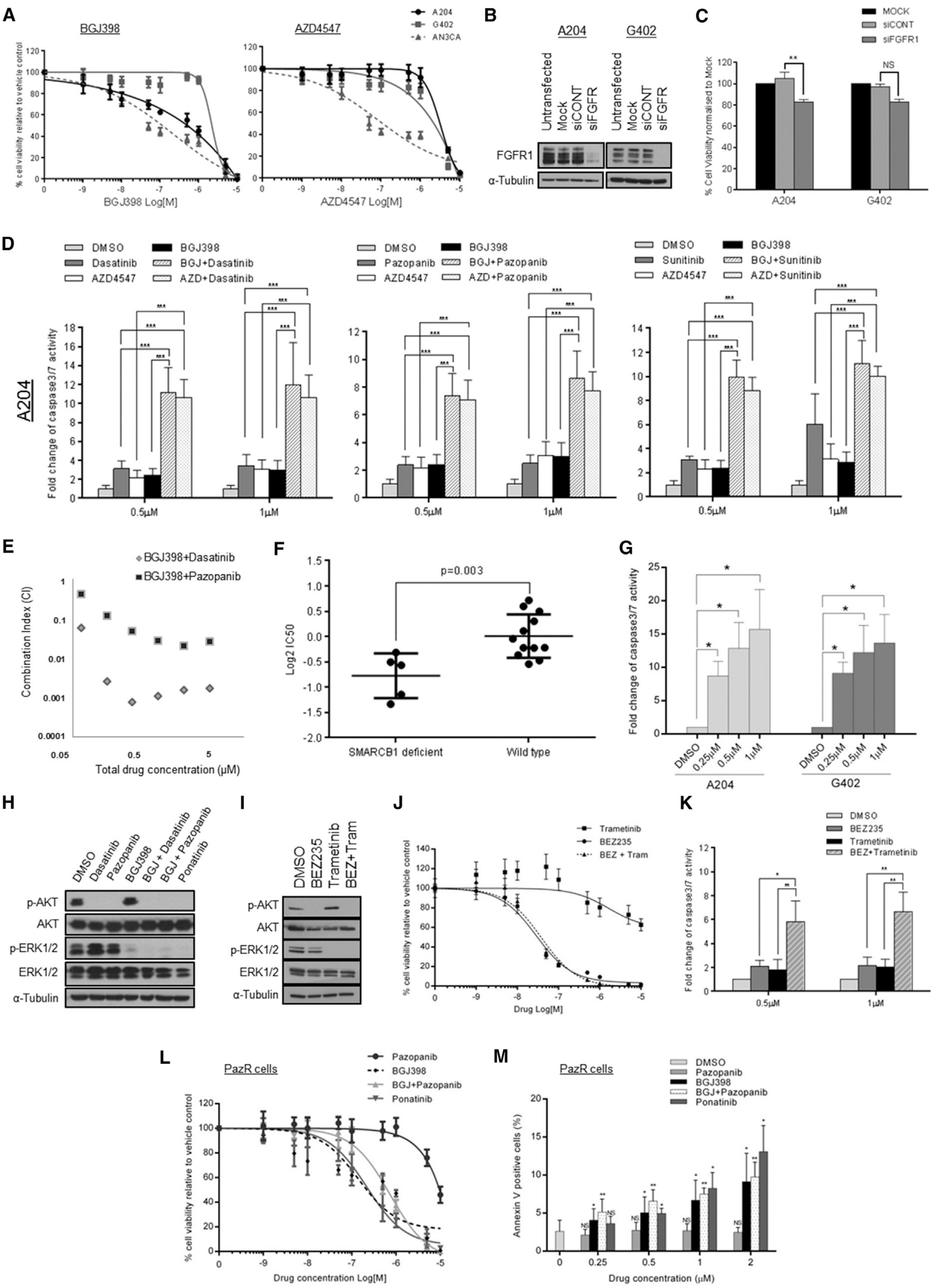

(legend on next page)

specimens composed of 12 MRTs and 11 AT/RTs. RNA-seq data from 172 normal tissue samples from the Illumina Bodymap were used as controls. Read count data in RT samples showed an average of 2,514 and 12,127 normalized reads mapped to *PDGFRA* and *FGFR1*, respectively, indicating moderate-high expression of each gene. Average levels of expression were significantly higher in RT than in the normal tissue collection for *PDGFRA* ($p = 0.016$) and *FGFR1* ($p < 0.001$). Indeed, a substantial number of RT samples (35% and 70% of primary RT samples for *PDGFRA* and *FGFR1*, respectively) showed greater expression than the 95th percentile of normal tissue samples for these genes, implying a large degree of tumorigenic overexpression (Figure 4A; Table S3).

Immunohistochemistry was performed on two MRT and two AT/RT cases to determine PDGFRα and FGFR1 protein expression levels (Figures 4B and S4). All cases showed no nuclear staining for SMARCB1. FGFR1 stained positively in the cytoplasm in three of four cases, and in both MRT specimens (NMB957 and NMB997) additional membrane staining was observed. PDGFRα was expressed in all four RT cases with cytoplasmic staining in tumor cells. As a comparison, we assessed two medulloblastoma cases (NMB361 and NMB795), which showed nuclear staining for SMARCB1 and were negative for both PDGFRα and FGFR1 (Figure 4B). These findings support the RNA-seq analysis and confirm that both PDGFRα and FGFR1 are expressed in RT patient specimens.

DISCUSSION

MRTs are often lethal within the first year of diagnosis and many patients are refractory to standard chemotherapy (Madigan et al., 2007). There is thus an urgent need for new effective therapies. This study demonstrates that dual inhibition of PDGFRα and FGFR1 leads to suppression of AKT and ERK1/2 phosphorylation, resulting in synergistic cytotoxicity in MRT cells. Previous reports have found that A204 cells are sensitive to sunitinib and dasatinib (albeit mislabeled as a rhabdomyosarcoma line) through the inhibition of PDGFRα (Bai et al., 2012; McDermott et al., 2009). The FGFR inhibitor BGJ398 also has been shown to reduce MRT cell growth (Wöhrle et al., 2013). However, our experiments find that these inhibitors have limited utility as single agents and do not induce apoptosis. Dual blockade of both RTKs promotes cytotoxicity across all drug doses tested in A204 and G402 cells. While TKI combinations may display better efficacy, there is a risk of greater toxicities. We show that ponatinib, a dual PDGFRα and FGFR1 inhibitor, induces apoptosis in MRT cells as a single agent. Given that our data document the expression of both PDGFRα and FGFR1 in RT patient specimens, we posit that ponatinib is a candidate for further evaluation in the treatment of RT patients. It should be noted that additional pre-clinical work in in vivo models is required to determine the therapeutic window of ponatinib or dual inhibition of PDGFRα and FGFR1 in order to take this therapeutic strategy further into clinical trials.

Our findings have implications for other cancer types that harbor SMARCB1 deficiencies, including epithelioid sarcomas, renal medullary carcinoma, epithelioid malignant peripheral nerve sheath tumors, and extraskeletal myxoid chondrosarcomas (Margol and Judkins, 2014). The SS18-SSX fusion in synovial sarcoma is known to disrupt the SWI/SNF assembly, resulting in SMARCB1-deficient complexes (Kadoch and Crabtree, 2013). Furthermore, reduced SMARCB1 protein expression is found in a proportion of synovial sarcomas (Margol and Judkins, 2014). Our data indicate that it may be beneficial to assess PDGFRα and FGFR1 levels to determine if ponatinib has similar efficacy in these cancers.

Mutations in SWI/SNF subunits are found in ~20% of cancers (Kadoch et al., 2013). It was reported recently that EGFR

Figure 3. Dual Inhibition of PDGFRα and FGFR1 Is Cytotoxic in MRT Cells

(A) Dose-response curves for MRT and AN3CA cell lines upon treatment with FGFR inhibitors BGJ398 and AZD4547. Cell viability is normalized to DMSO control (n = 3).

(B) Immunoblot of FGFR1 expression in MRT cells under mock, non-targeting control siCONT and siFGFR1 pool transfection conditions is shown.

(C) Bar plots showing cell viability of MRT cells upon siRNA silencing of FGFR1. Cell viability data are normalized to mock transfection (n = 3). Statistical analysis of siFGFR1 versus siCONT was performed by paired Student's t test (**$p < 0.01$; NS, not significant).

(D) Bar plots showing the normalized fold change in caspase 3/7 activity in the A204 cells treated with PDGFRα and FGFR inhibitors or a combination at the indicated doses (n = 3). Data for G402 cells are presented in Figure S2B. Data are normalized to DMSO control. Statistical analysis between combination and single TKI treatment was done by ANOVA with Tukey's multiple comparison test (***$p < 0.001$).

(E) Combination index (CI) measurements for BGJ398 and PDGFRα inhibitors in A204 cells show synergy (CI < 1) across all doses tested. Individual dose-response measurements are presented in Figure S2D.

(F) Log_2 IC_{50} values of SMARCB1-deficient (n = 5) versus wild-type (n = 12) cell lines in response to ponatinib treatment. A panel of 17 cell lines was treated with a range of ponatinib concentrations. Cell viability is normalized to DMSO control (n = 2). Statistical analysis was performed by paired Student's t test.

(G) Bar plots showing the normalized fold change in caspase 3/7 activity in the A204 and G402 cells treated with ponatinib (n = 3). Data are normalized to DMSO control. Statistical analysis was performed by paired Student's t test (*$p < 0.05$).

(H) Immunoblot of AKT and ERK1/2 phosphorylation levels in A204 cells upon drug treatment with TKI at the 1 μM dose for 1 hr is shown.

(I) Immunoblot of AKT and ERK1/2 phosphorylation levels in A204 cells upon drug treatment with BEZ235 and trametinib at the 1 μM dose for 1 hr is shown.

(J) Dose-response curves for A204 cells treated with BEZ235, trametinib, and a combination of both. Cell viability is normalized to DMSO control (n = 3).

(K) Bar plots showing the normalized fold change in caspase 3/7 activity in the A204 cells treated with BEZ235, trametinib, or a combination at the indicated doses (n = 3). Statistical analysis between combination and single kinase inhibitor treatment was performed by paired Student's t test (*$p < 0.05$ and **$p < 0.01$).

(L) Dose-response curves for PazR cells treated with pazopanib, BGJ398, a combination of both, or ponatinib. Cell viability is normalized to DMSO control (n = 3). IC_{50} values are detailed in Table S2.

(M) Bar plots showing percentage of annexin V staining in PazR cells treated with pazopanib, BGJ398, a combination of both inhibitors, or ponatinib (n = 3). Statistical analysis of TKI treatment versus DMSO was done by paired Student's t test (*$p < 0.05$ and **$p < 0.01$; NS, not significant).

Data presented for (A), (C), (D), (F), (G), (J), (K), (L), and (M) are means ± SD.

OPEN
ACCESS
CellPress

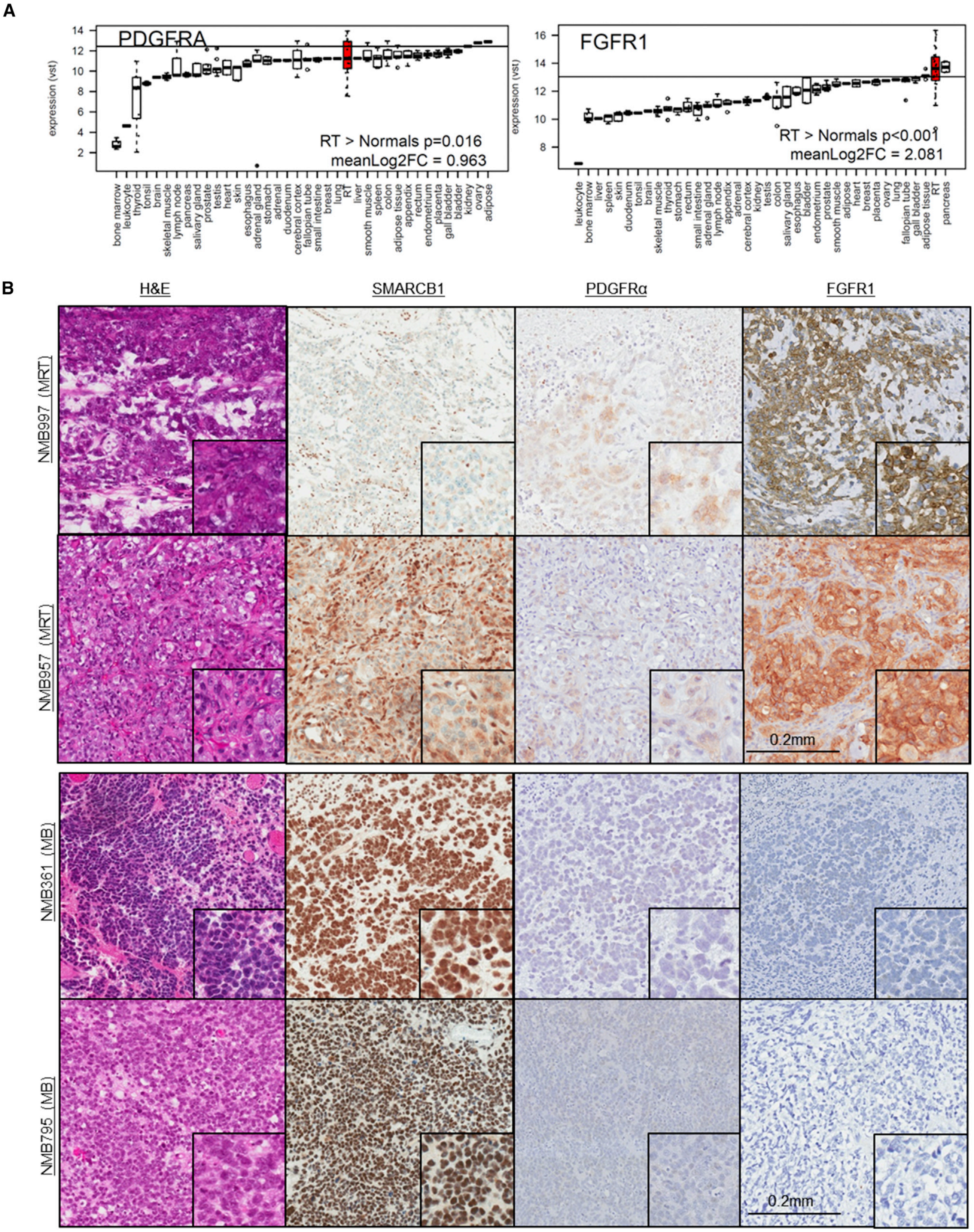

(legend on next page)

expression is regulated by SMARCE1, SMARCA4, and ARID1A and that SMARCE1 deficiency confers TKI resistance in lung cancer (Papadakis et al., 2015). Wöhrle et al. (2013) showed that FGFR1 is upregulated when SMARCB1 is deleted in MRT cells, while Darr et al. (2015) recently demonstrated that EGFR expression is regulated by SMARCB1. In our experiments, ectopic expression of SMARCB1 in MRT cells show that FGFR1 and EGFR are only regulated by SMARCB1 in the A204 cells and not the G402 line (Figure S2G), suggesting that there may be some context specificity associated with the regulation of RTK expression levels by SMARCB1. By showing that SMARCB1 loss also regulates PDGFRα expression levels, our study provides further evidence that exploiting RTK dependencies in cancers driven by SWI/SNF deficiencies is an effective therapeutic strategy in vitro. Since it is currently not possible to directly target the SWI/SNF complex, TKI combinations may have broader clinical utility in the treatment of this class of cancers.

Acquired resistance and tumor recurrence is common in patients undergoing TKI therapy. Pazopanib is approved for sarcoma treatment, but patients eventually develop resistance by mechanisms that are unknown (Kasper et al., 2014). Our study presents one mechanism of acquired resistance to pazopanib in soft tissue malignancies through PDGFRα loss and bypass of the AKT-signaling pathway, and it provides a means to overcome this resistance via FGFR1 blockade in vitro. Since it is less likely for cancer cells to develop acquired resistance when multiple RTKs are simultaneously inhibited up front, there is a rationale for using the PDGFRα and FGFR1 inhibitor combination as first-line therapy (Tan et al., 2016). Indeed, attempts by our laboratory to generate acquired resistant lines to the PDGFRα and FGFR inhibitor combination have been unsuccessful (Figure S2H). Determination of FGFR1 levels in patients who develop resistance to pazopanib may stratify cases that could benefit from subsequent therapy with FGFR inhibitors. There remain several outstanding questions that need to be addressed in future studies, such as specifically how PDGFRα is downregulated in the PazR cells and what the mechanism is by which FGFR1 blockade overcomes resistance. Phosphoproteomic analysis of the PazR cells revealed candidate pathways such as PLCG1 and Src family kinases (YES1, FYN, and FGR), which are upregulated. These proteins serve as targets for future evaluation as additional means to overcome acquired pazopanib resistance.

In summary, we show that MRTs are exquisitely sensitive to the combined inhibition of PDGFRα and FGFR1 and that ponatinib is effective as a single agent in this disease in the in vitro setting. A previous chemical inhibitor screen in AT/RT cell lines found that the PDGFRα and FGFR inhibitors were not effective in reducing cell viability (Singh et al., 2013), highlighting the complexity of cell-type-specific signaling dependencies in SMARCB1-deficient cell lines. Given the recent identification of distinct epigenetic subgroups in AT/RTs (Johann et al., 2016), future work will need to establish if PDGFRα and FGFR dependencies are linked to specific molecular subgroups in SMARCB1-deficient tumors. We also find that treatment with FGFR inhibitors sensitizes MRT cells that have acquired resistance to pazopanib. This study provides proof-of-principle that exploiting RTK co-activation dependencies may have utility in the treatment of cancers with deficiencies in SWI/SNF subunits.

EXPERIMENTAL PROCEDURES

Cell Culture

A204, G402, and G401 cells were obtained from ATCC. CHLA226 and BT12 cells were provided by the Children's Oncology Group Cell Culture Repository. All other lines were a gift from Dr. Janet Shipley. Details for cell culture conditions and derivation of acquired resistant sublines are described in the Supplemental Experimental Procedures.

Molecular Biology and Lentiviral Infection

The procedure for ectopic expression of SMARCB1 by lentiviral infection is detailed in the Supplemental Experimental Procedures.

Immunoblotting, Immunoprecipitation, and Immunofluorescence

After the indicated treatments, cells were lysed in radio-immunoprecipitation assay (RIPA) lysis buffer at 4°C. Lysates were loaded onto SDS-PAGE gels followed by blotting onto polyvinylidene fluoride (PVDF) membranes. Details of antibodies and immunoprecipitation and immunofluorescence analyses are presented in the Supplemental Experimental Procedures.

Cell Viability and Apoptosis Assays

Cells (2,000/well) were seeded in a 96-well plate, and they were treated with inhibitors at the indicated dose and combinations for 24 hr for apoptosis measurement by Caspase-Glo 3/7 Assay (Promega) or for 72 hr in cell viability measurements by WST-1 (Abcam), following the manufacturer's recommendations. IC_{50} data were generated from dose-response curves fitted using a four-parameter regression fit in PRISM 5 software (GraphPad). Details for annexin V staining and siRNA transfections are given in the Supplemental Experimental Procedures.

aCGH, Gene Expression, and Phosphoproteomic Analysis

Genomic DNA was extracted and analyzed on an in-house aCGH platform consisting of ~32,000 bacterial artificial clones (BACs) tiled across the genome. Descriptions of platform and data analysis procedure are provided in the Supplemental Experimental Procedures. RNA was extracted and gene expression analysis was performed on Illumina HTv12 chip as per the manufacturer's recommendations. Data analysis methodology is presented in the Supplemental Experimental Procedures. Phosphoproteomic analysis was performed as described (Iwai et al., 2013), with the modifications in protocol and bioinformatic analysis as detailed in the Supplemental Experimental Procedures.

RNA-Seq and Immunohistochemistry of Patient Specimens

Human tumor samples were provided by the UK CCLG as part of CCLG-approved biological study (2012 BS 05). Informed consent was obtained

Figure 4. PDGFRα and FGFR1 Are Expressed in Rhabdoid Tumor Patient Specimens

(A) Relative RNA expression levels of *PDGFRA* and *FGFR1* genes between rhabdoid tumors (RTs) and various normal tissues. Boxplots show log2 variance-stabilized transformed expression across the samples. Boxplots show median score (thick black line), interquartile ranges (extent of box), and range (whiskers). Boxplots are ordered according to median expression level and RT is highlighted in red. The p values indicate one-sided t test between RT and normal tissues. The horizontal line represents the 95th percentile of all normal tissues for each gene. RNA expression data are provided in Table S3.

(B) Immunohistochemical analysis of MRT and medulloblastoma (MB) patient specimens for H&E, anti-SMARCB1, anti-PDGFRα, and anti-FGFR1 staining. Scale bar represents 0.2 mm.

from all subjects. Human tumor investigations were conducted with approval from Newcastle/North Tyneside Research Ethics Committee (study reference 07/Q0905/71). RNA was extracted from 23 fresh frozen tumor tissue samples taken from pediatric patients with a confirmed diagnosis of *SMARCB1* negative RT. A paired-end cDNA sequencing library was created and sequenced on an Illumina Hi-Seq2500 to give ~90 M paired-end reads. Reads were quality checked, aligned, and normalized gene counts were generated using Gencodev19 Transcriptome library. Variance stabilizing transformations of normalized counts were used as a measure of gene expression (full details are given in the Supplemental Experimental Procedures). RNA-seq data from normal tissues were taken from Illumina Bodymap (ArrayExpress: E-MTAB-513 and E-MTAB-2836). Full details for immunohistochemistry analysis are provided in the Supplemental Experimental Procedures.

Statistical Methods

Experimental results are representative of at least three independent experiments. The statistical significance of data in all figures was evaluated by Student's t test. Statistical analysis between combination and single treatment (Figure 3D) was done by ANOVA with Tukey's multiple comparison test. Calculations were performed with GraphPad Prism software.

ACCESSION NUMBERS

The accession numbers for the microarray gene expression data and the array CGH data reported in this paper are GEO: GSE78864 and GSE87208, respectively.

SUPPLEMENTAL INFORMATION

Supplemental Information includes Supplemental Experimental Procedures, four figures, and three tables and can be found with this article online at http://dx.doi.org/10.1016/j.celrep.2016.10.005.

AUTHOR CONTRIBUTIONS

Conceptualization, P.H.H.; Methodology, J.P.W., J.R.T., and P.H.H.; Investigation, J.P.W., J.R.T., M.A.F., F.M., M.B., S.V., M.T.L., K.H., L.S.P., P.W., K.A.R., A.J., F.D., S.C., B.T., A.-C.T., R.C.N., and D.W.; Resources, M.A.F., S.C., A.-C.T., R.C.N., and D.W.; Writing – Original Draft, J.P.W. and P.H.H.; Writing – Review & Editing, M.A.F., A.-C.T, R.C.N., D.W., and P.H.H.; Funding Acquisition, A.-C.T., R.C.N., D.W., and P.H.H.

ACKNOWLEDGMENTS

This work was supported by grants from the Institute of Cancer Research (ICR), Cancer Research UK (C36478/A19281), Sarcoma UK (003.2014), Royal Marsden Cancer Charity, and Biotechnology and Biological Sciences Research Council (BBSRC) (BB/I014276/I and BB/M013782/1) to P.H.H.; Breast Cancer Now (SF01May2011) to R.C.N.; NIH (T32CA17468) to K.A.R.; David F. and Margaret T. Grohne Family Foundation to A.-C.T.; and Children With Cancer UK (2012/134), North Eastern Children's Cancer Research (NECCR) (NCC Core Grant Support) to D.W., whose work was undertaken within the remit of a Children's Cancer and Leukaemia Group (CCLG) Biological Study 2012 BS 05 and as part of the INSTINCT network programme grant, co-funded by The Brain Tumour Charity, Great Ormond Street Children's Charity, and Children With Cancer UK (16/193). The CHLA226 and BT12 cell lines were kindly provided by the Children's Oncology Group Cell Culture Repository.

Received: March 4, 2016
Revised: August 7, 2016
Accepted: September 30, 2016
Published: October 25, 2016

REFERENCES

Anastassiadis, T., Deacon, S.W., Devarajan, K., Ma, H., and Peterson, J.R. (2011). Comprehensive assay of kinase catalytic activity reveals features of kinase inhibitor selectivity. Nat. Biotechnol. *29*, 1039–1045.

Bai, Y., Li, J., Fang, B., Edwards, A., Zhang, G., Bui, M., Eschrich, S., Altiok, S., Koomen, J., and Haura, E.B. (2012). Phosphoproteomics identifies driver tyrosine kinases in sarcoma cell lines and tumors. Cancer Res. *72*, 2501–2511.

Chun, H.J., Lim, E.L., Heravi-Moussavi, A., Saberi, S., Mungall, K.L., Bilenky, M., Carles, A., Tse, K., Shlafman, I., Zhu, K., et al. (2016). Genome-wide profiles of extra-cranial malignant rhabdoid tumors reveal heterogeneity and dysregulated developmental pathways. Cancer Cell *29*, 394–406.

Darr, J., Klochendler, A., Isaac, S., Geiger, T., and Eden, A. (2015). Phosphoproteomic analysis reveals Smarcb1 dependent EGFR signaling in Malignant Rhabdoid tumor cells. Mol. Cancer *14*, 167.

Davis, M.I., Hunt, J.P., Herrgard, S., Ciceri, P., Wodicka, L.M., Pallares, G., Hocker, M., Treiber, D.K., and Zarrinkar, P.P. (2011). Comprehensive analysis of kinase inhibitor selectivity. Nat. Biotechnol. *29*, 1046–1051.

Frühwald, M.C., Biegel, J.A., Bourdeaut, F., Roberts, C.W., and Chi, S.N. (2016). Atypical teratoid/rhabdoid tumors-current concepts, advances in biology, and potential future therapies. Neuro-oncol. *18*, 764–778.

Gozgit, J.M., Wong, M.J., Wardwell, S., Tyner, J.W., Loriaux, M.M., Mohemmad, Q.K., Narasimhan, N.I., Shakespeare, W.C., Wang, F., Druker, B.J., et al. (2011). Potent activity of ponatinib (AP24534) in models of FLT3-driven acute myeloid leukemia and other hematologic malignancies. Mol. Cancer Ther. *10*, 1028–1035.

Hata, A.N., Niederst, M.J., Archibald, H.L., Gomez-Caraballo, M., Siddiqui, F.M., Mulvey, H.E., Maruvka, Y.E., Ji, F., Bhang, H.E., Krishnamurthy Radhakrishna, V., et al. (2016). Tumor cells can follow distinct evolutionary paths to become resistant to epidermal growth factor receptor inhibition. Nat. Med. *22*, 262–269.

Huang, P.H., Mukasa, A., Bonavia, R., Flynn, R.A., Brewer, Z.E., Cavenee, W.K., Furnari, F.B., and White, F.M. (2007). Quantitative analysis of EGFRvIII cellular signaling networks reveals a combinatorial therapeutic strategy for glioblastoma. Proc. Natl. Acad. Sci. USA *104*, 12867–12872.

Iwai, L.K., Payne, L.S., Luczynski, M.T., Chang, F., Xu, H., Clinton, R.W., Paul, A., Esposito, E.A., Gridley, S., Leitinger, B., et al. (2013). Phosphoproteomics of collagen receptor networks reveals SHP-2 phosphorylation downstream of wild-type DDR2 and its lung cancer mutants. Biochem. J. *454*, 501–513.

Johann, P.D., Erkek, S., Zapatka, M., Kerl, K., Buchhalter, I., Hovestadt, V., Jones, D.T., Sturm, D., Hermann, C., Segura Wang, M., et al. (2016). Atypical teratoid/rhabdoid tumors are comprised of three epigenetic subgroups with distinct enhancer landscapes. Cancer Cell *29*, 379–393.

Kadoch, C., and Crabtree, G.R. (2013). Reversible disruption of mSWI/SNF (BAF) complexes by the SS18-SSX oncogenic fusion in synovial sarcoma. Cell *153*, 71–85.

Kadoch, C., Hargreaves, D.C., Hodges, C., Elias, L., Ho, L., Ranish, J., and Crabtree, G.R. (2013). Proteomic and bioinformatic analysis of mammalian SWI/SNF complexes identifies extensive roles in human malignancy. Nat. Genet. *45*, 592–601.

Kasper, B., Sleijfer, S., Litière, S., Marreaud, S., Verweij, J., Hodge, R.A., Bauer, S., Kerst, J.M., and van der Graaf, W.T. (2014). Long-term responders and survivors on pazopanib for advanced soft tissue sarcomas: subanalysis of two European Organisation for Research and Treatment of Cancer (EORTC) clinical trials 62043 and 62072. Ann. Oncol. *25*, 719–724.

Kim, K.H., and Roberts, C.W. (2014). Mechanisms by which SMARCB1 loss drives rhabdoid tumor growth. Cancer Genet. *207*, 365–372.

Lee, R.S., Stewart, C., Carter, S.L., Ambrogio, L., Cibulskis, K., Sougnez, C., Lawrence, M.S., Auclair, D., Mora, J., Golub, T.R., et al. (2012). A remarkably simple genome underlies highly malignant pediatric rhabdoid cancers. J. Clin. Invest. *122*, 2983–2988.

Lemmon, M.A., and Schlessinger, J. (2010). Cell signaling by receptor tyrosine kinases. Cell *141*, 1117–1134.

Madigan, C.E., Armenian, S.H., Malogolowkin, M.H., and Mascarenhas, L. (2007). Extracranial malignant rhabdoid tumors in childhood: the Childrens Hospital Los Angeles experience. Cancer *110*, 2061–2066.

Margol, A.S., and Judkins, A.R. (2014). Pathology and diagnosis of SMARCB1-deficient tumors. Cancer Genet. *207*, 358–364.

McDermott, U., Ames, R.Y., Iafrate, A.J., Maheswaran, S., Stubbs, H., Greninger, P., McCutcheon, K., Milano, R., Tam, A., Lee, D.Y., et al. (2009). Ligand-dependent platelet-derived growth factor receptor (PDGFR)-alpha activation sensitizes rare lung cancer and sarcoma cells to PDGFR kinase inhibitors. Cancer Res. *69*, 3937–3946.

Papadakis, A.I., Sun, C., Knijnenburg, T.A., Xue, Y., Grernrum, W., Hölzel, M., Nijkamp, W., Wessels, L.F., Beijersbergen, R.L., Bernards, R., and Huang, S. (2015). SMARCE1 suppresses EGFR expression and controls responses to MET and ALK inhibitors in lung cancer. Cell Res. *25*, 445–458.

Singh, A., Lun, X., Jayanthan, A., Obaid, H., Ruan, Y., Strother, D., Chi, S.N., Smith, A., Forsyth, P., and Narendran, A. (2013). Profiling pathway-specific novel therapeutics in preclinical assessment for central nervous system atypical teratoid rhabdoid tumors (CNS ATRT): favorable activity of targeting EGFR- ErbB2 signaling with lapatinib. Mol. Oncol. *7*, 497–512.

Szerlip, N.J., Pedraza, A., Chakravarty, D., Azim, M., McGuire, J., Fang, Y., Ozawa, T., Holland, E.C., Huse, J.T., Jhanwar, S., et al. (2012). Intratumoral heterogeneity of receptor tyrosine kinases EGFR and PDGFRA amplification in glioblastoma defines subpopulations with distinct growth factor response. Proc. Natl. Acad. Sci. USA *109*, 3041–3046.

Tan, L., Wang, J., Tanizaki, J., Huang, Z., Aref, A.R., Rusan, M., Zhu, S.J., Zhang, Y., Ercan, D., Liao, R.G., et al. (2014). Development of covalent inhibitors that can overcome resistance to first-generation FGFR kinase inhibitors. Proc. Natl. Acad. Sci. USA *111*, E4869–E4877.

Tan, A.C., Vyse, S., and Huang, P.H. (2016). Exploiting receptor tyrosine kinase co-activation for cancer therapy. Drug Discov. Today, S1359-6446(16)30260-4.

Tucker, J.A., Klein, T., Breed, J., Breeze, A.L., Overman, R., Phillips, C., and Norman, R.A. (2014). Structural insights into FGFR kinase isoform selectivity: diverse binding modes of AZD4547 and ponatinib in complex with FGFR1 and FGFR4. Structure *22*, 1764–1774.

Wöhrle, S., Weiss, A., Ito, M., Kauffmann, A., Murakami, M., Jagani, Z., Thuery, A., Bauer-Probst, B., Reimann, F., Stamm, C., et al. (2013). Fibroblast growth factor receptors as novel therapeutic targets in SNF5-deleted malignant rhabdoid tumors. PLoS ONE *8*, e77652.

Xu, A.M., and Huang, P.H. (2010). Receptor tyrosine kinase coactivation networks in cancer. Cancer Res. *70*, 3857–3860.

Cell Reports, Volume *17*

Supplemental Information

Dual Targeting of PDGFRα and FGFR1 Displays Synergistic Efficacy in Malignant Rhabdoid Tumors

Jocelyn P. Wong, Jason R. Todd, Martina A. Finetti, Frank McCarthy, Malgorzata Broncel, Simon Vyse, Maciej T. Luczynski, Stephen Crosier, Karen A. Ryall, Kate Holmes, Leo S. Payne, Frances Daley, Patty Wai, Andrew Jenks, Barbara Tanos, Aik-Choon Tan, Rachael C. Natrajan, Daniel Williamson, and Paul H. Huang

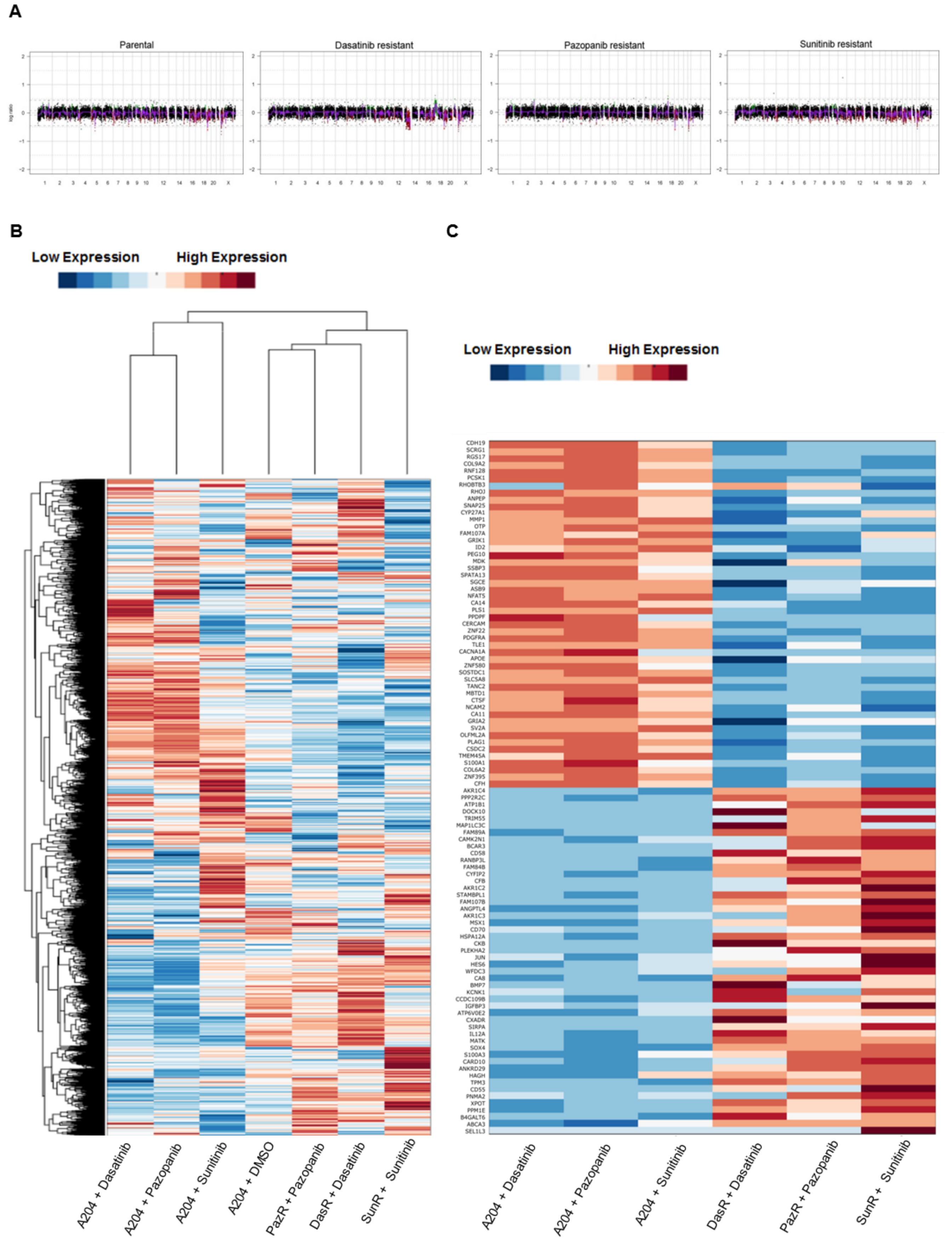

Figure S1. ***Related to Figure 2*** (A) Microarray-based comparative genomic hybridisation plots of A204 parental and resistant cells displaying the full genomic profiles of the four cell lines. (B) Hierarchical clustering of gene expression dataset of parental A204 cells treated with DMSO control or each of the three PDGFRα TKIs and each resistant subline treated with their respective TKI. DasR=dasatinib resistant, PazR=pazopanib resistant and SunR=sunitinib resistant. (C) Heatmap of the top 50 upregulated and downregulated genes in the resistant sublines versus the parental A204 cells treated with TKIs.

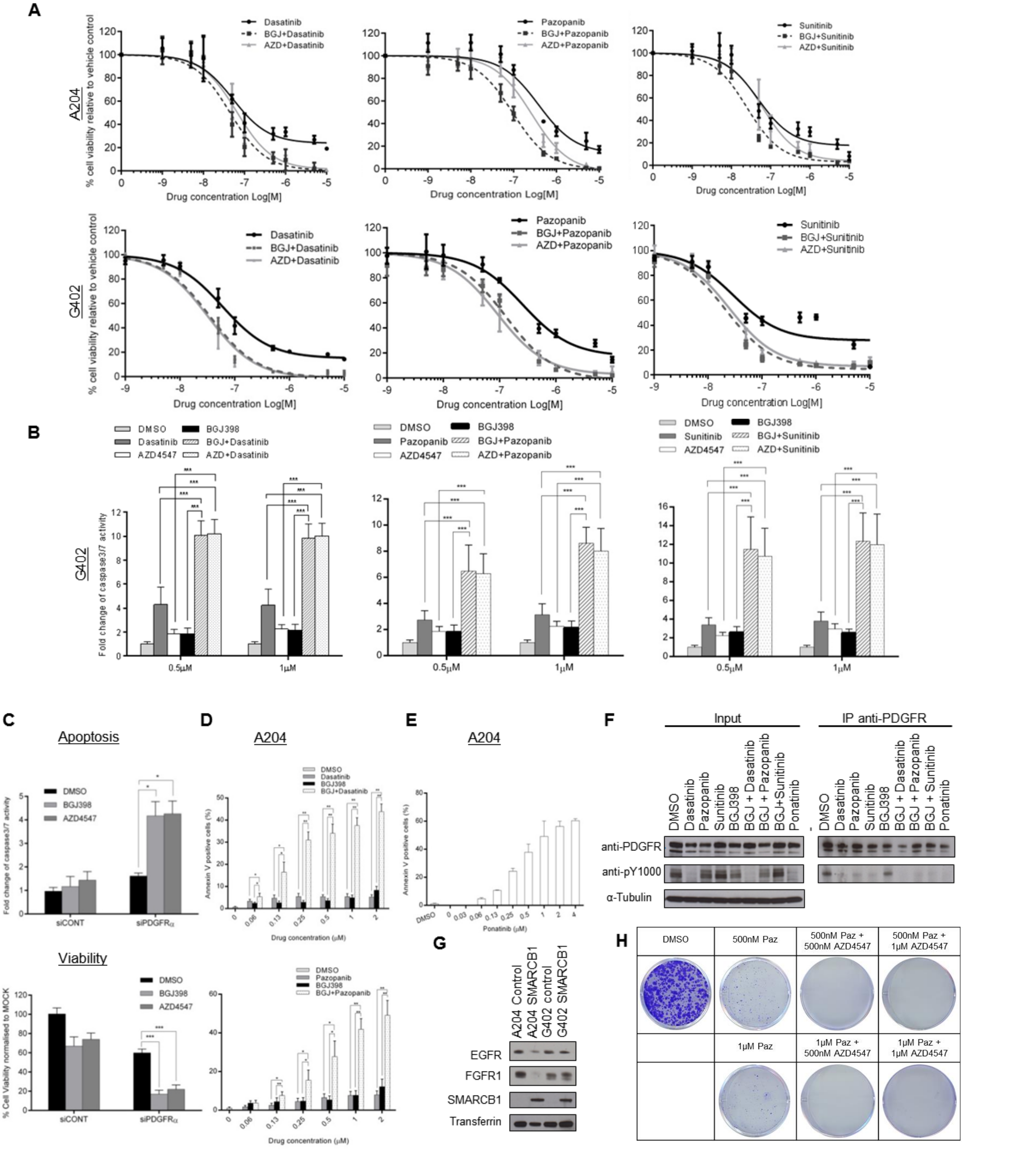

Figure S2. ***Related to Figure 3*** Dual inhibition of PDGFRα and FGFR1 is cytotoxic in MRT cells. (A) Dose response curves for A204 and G402 cells upon treatment with PDGFRα and a combination of PDGFRα and FGFR inhibitors. Cell viability data is normalised to DMSO control (n=3). (B) Bar plots showing the normalised fold change in caspase 3/7 activity in the G402 cells upon treatment with PDGFRα and FGFR inhibitors or a combination at the indicated doses (n=3). Data is normalised to DMSO control. Statistical significance of combination versus single TKI treatment was performed by ANOVA with Tukey's multiple comparisons test where ***$p<0.001$. (C) Bar plots showing apoptosis measured by caspase 3/7 activity (upper panel) and viability (lower panel) of A204 cells treated with FGFR inhibitors in combination with siRNA depletion of PDGFRα. Statistical analysis of FGFR inhibitors versus DMSO control was performed by paired Student's t test where *$p<0.05$ and ***$p<0.001$. (D) Bar plots showing percentage Annexin V staining in A204 parental cells when treated with PDGFRα inhibitor, BGJ398 or a combination of both inhibitors (n=3) where *$p<0.05$ and **$p<0.01$. (E) Bar plots showing percentage Annexin V staining in A204 parental cells treated with ponatinib (n=3). (F) Immunoprecipitation of PDGFRα followed by immunoblotting with phosphotyrosine-specific antibody (PY1000) in A204 cells upon treatment with 1μM PDGFRα inhibitor, BGJ398, combination or ponatinib for 1 hour. (G) Immunoblot of FGFR1 and EGFR showing modulation of receptor levels upon ectopic SMARCB1 expression in A204 and G402 cells. (H) Colony formation assay showing that pazopanib treatment over 2 weeks leads to resistant colony formation in the A204 cells. However treatment with high dose combination of pazopanib and AZD4547 led to no colonies, providing support that first line combination therapy prevents acquisition of resistance. For (A), (B), (C), (D) and (E), all values are mean ± SD.

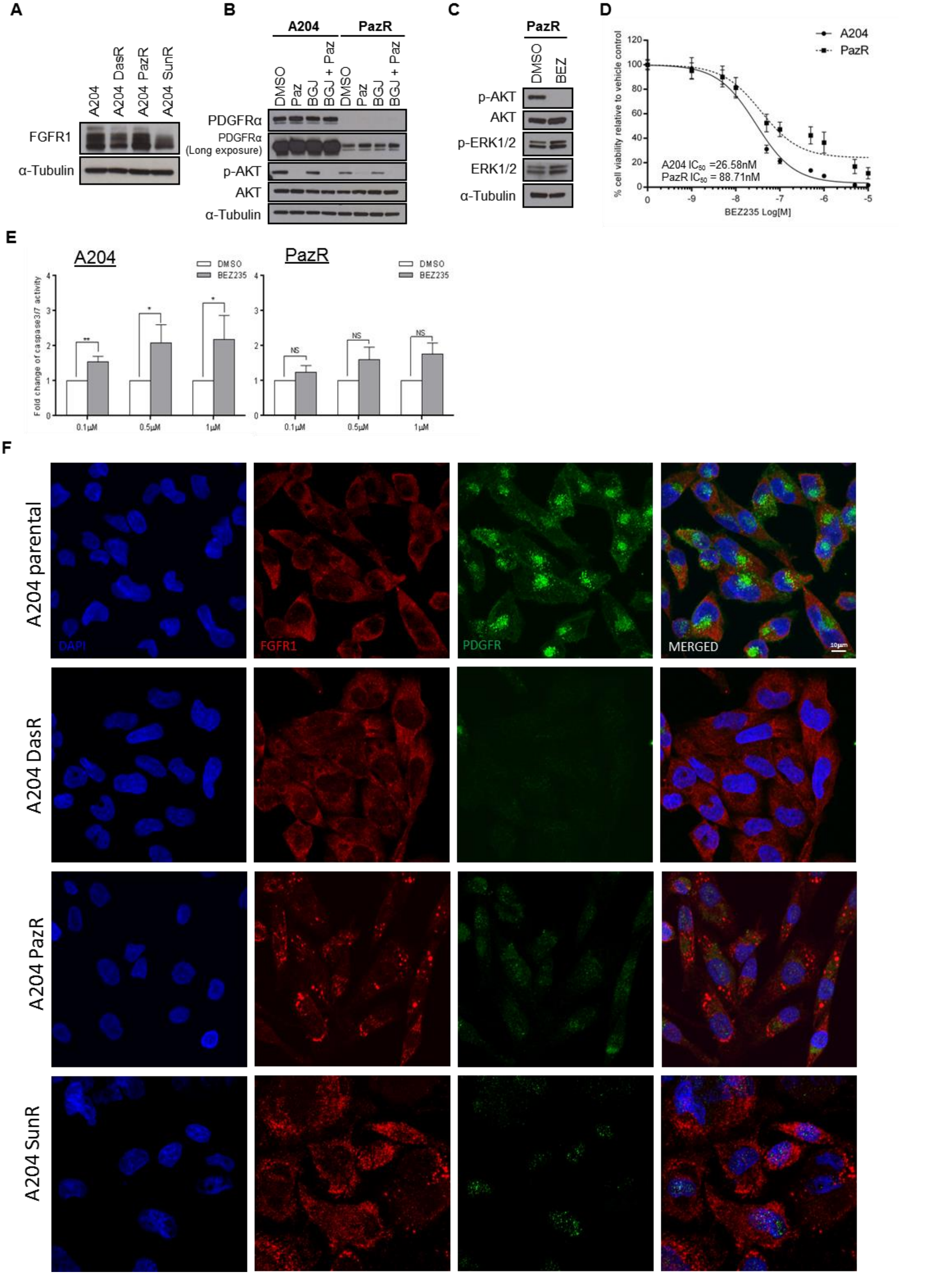

Figure S3. ***Related to Figure 3*** Targeting FGFR1 sensitizes acquired resistance to pazopanib. (A) Immunoblot of FGFR1 expression in the parental A204 and resistant sublines. DasR=dasatinib resistant, PazR=pazopanib resistant and SunR=sunitinib resistant. (B) Immunoblot of PDGFRα levels and AKT pathway activation in the parental A204 and PazR resistant cells upon treatment with 1μM pazopanib, BGJ398 or a combination for 1 hour. (C) Immunoblot of AKT and ERK1/2 phosphorylation levels in PazR cells upon treatment with 1μM BEZ235 for 1 hour. (D) Dose response curves for A204 and PazR cells upon treatment with BEZ235. Cell viability data is normalised to DMSO control (n=3). Values are mean ± SD. (E) Bar plots showing the normalised fold change in caspase 3/7 activity in the A204 (left) and PazR (right) cells upon treatment with BEZ235 at the indicated doses (n=3). Data is normalised to DMSO control. Statistical significance of DMSO versus BEZ235 treatment was performed by paired Student's t test where *p<0.05 and NS is not significant. (F) Representative images of dual-colour immunofluorescence analysis of parental A204 and resistant sublines, DAPI (blue), FGFR1 (red) and PDGFRα (green) showing that FGFR1 and PDGFRα expression is uniformly distributed in all cells within the parental A204 population.

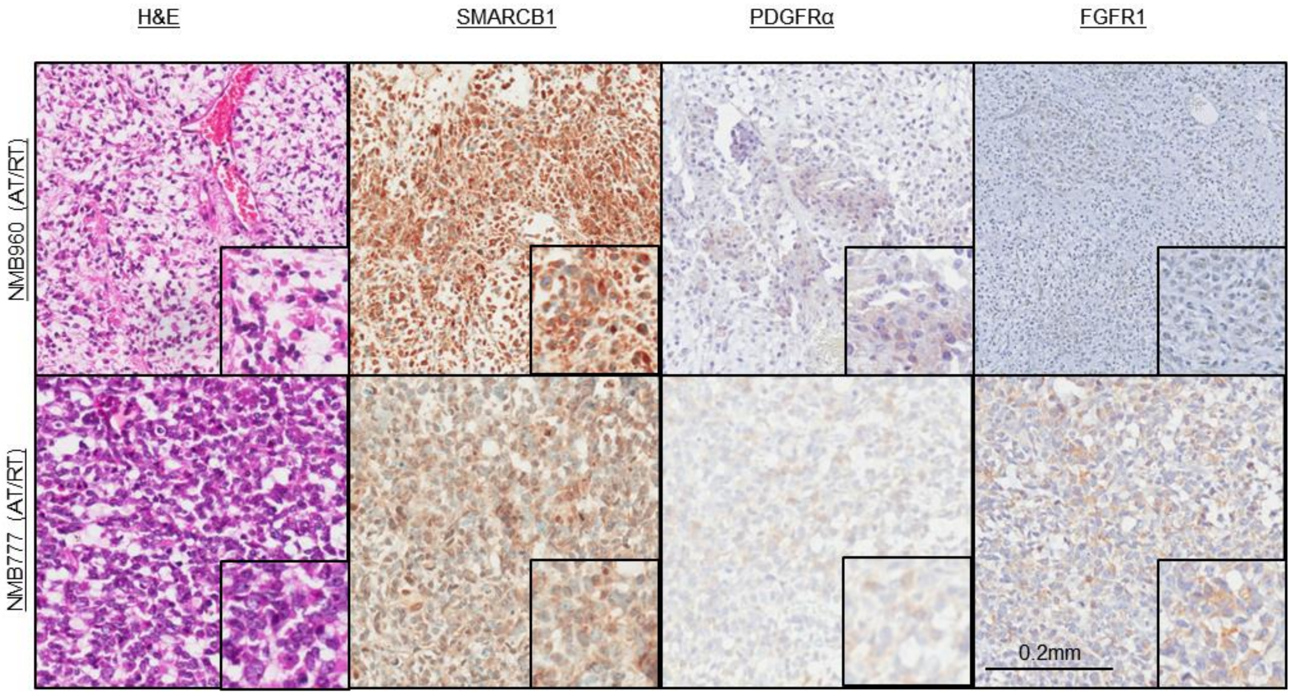

Figure S4. ***Related to Figure 4*** Immunohistochemical analysis of AT/RT patient specimens for haematoxylin and eosin (H&E), anti-SMARCB1, anti-PDGFRα and anti-FGFR1 staining. Scale bar represents 0.2mm.

Table S1. Dasatinib, Pazopanib, Sunitinib IC50 concentrations in a panel of 14 cell lines and A204 resistant cell lines .

Related to Figure 1.

Cell Line	Dasatinib IC50 (nM)	Pazopanib IC50 (nM)	Sunitinib IC50 (nM)
SAOS2	1152.3 +/- 311.0	>10000	4569.7 +/- 516.2
U2OS	>10000	>10000	>10000
HT1080	>10000	>10000	>10000
MES-SA	>10000	>10000	>10000
SJSA-1	>10000	>10000	>10000
SW684	62.4 +/- 25.9	>10000	>10000
SW872	1038 +/- 490.7	>10000	>10000
SW982	188.3 +/- 64.7	>10000	581.6 +/- 117.0
Hs729T	>10000	>10000	>10000
RMS-YM	>10000	>10000	>10000
RUCH-3	>10000	>10000	>10000
T91-95	>10000	>10000	>10000
G402	62.3 +/- 21.5	237.85 +/- 65.1	36.9 +/- 26.5
A204	41.8 +/- 5.1	218.7 +/- 19.6	36.3 +/- 5.5
A204 DasR	>10000	>10000	5010.7+/- 236.7
A204 PazR	>10000	>10000	>10000
A204 SunR	>10000	>10000	>10000

Table S2. Single and combination drug treatment IC50 concentrations in Pazopanib resistant A204 cell line.
Related to Figure 3.

Drug treatment	IC50 (nM)
Pazopanib	>10000
BGJ398	247.4 +/- 29.2
BGJ398 + Pazopanib	690.1 +/- 133.1
Ponatinib	271.5 +/- 167.8

Table S3. Variance stabilised RNASeq data of FGFR1 and PDGFRA.
Related to Figure 4.
(Excel File)

Supplemental Experimental Procedures

Cell culture and derivation of acquired resistant sublines

Cells were cultured in DMEM (A204, G402, Saos2, U2OS, HT1080, SW684, SW872, SW982, Hs729T, RUCH-3, T9195, BT12, CHLA266 and AN3CA), RPMI (G401, RMS-YM and SJSA-1) or McCoy5A (MES-SA) media supplemented with 10% FBS/2mM glutamine/100units/ml penicillin/100mg/ml streptomycin in 95% air/5% CO_2 atmosphere at 37°C. For SILAC experiments, A204 cells and resistant sublines were cultured in SILAC DMEM media (Thermo Fisher Scientific) supplemented with light lysine and arginine (R0K0) (Sigma) and heavy lysine and arginine (R10K8) (Goss Scientific) respectively.

Dasatinib, Pazopanib and Sunitinib (LC laboratories) were used to induce resistance in the A204 cells. Cells were grown initially in DMEM media containing drug concentration of 500nM. The drug was incremented when the cells had proliferated to near confluency alongside minimal visible cell death. Drug concentration was incremented from 2µM, 3µM and 5µM in a stepwise manner over 6 weeks. A final drug concentration of 5µM was maintained in resistant cells. Media and drug were replenished twice weekly.

Molecular biology and lentiviral infection

The pCDH-EF1-PURO-SMARCB1 plasmid was produced by PCR amplifying the whole SMARCB1 coding sequence from pCDNA 3.1-SMARCB1 (a gift from Frederique Quignon, Institute Curie). Restriction sites for XbaI and BamH1 were added to the Forward and Reverse primers respectively. The PCR product was digested and directionally ligated into the multiple cloning site of pCDH-EF1-Puro (Systems Biosciences).

PCDH-CMV-MCS-EF1-SMARCB1 Puro plasmid (System Bioscences) was transiently transfected into HEK293T cells using Calcium Phosphate Transfection method (CalPhos Transfection Kits, Clontech) according to manufacturer's instructions. Lentiviral infection of rhabdoid cells was carried out aiming to transduce about 60%-80% of the total amount of cells in each experiment, using an MOI of 10. To select for infected cells, Puromycin (Invitrogen) was added to the media to a final concentration of 1µg/mL for 72 hours prior to cell lysis.

Immunoblotting, immunoprecipitation and immunofluorescence

For immunoblotting, cells were lysed in RIPA lysis buffer supplemented with protease and phosphatase inhibitors (Thermo Pierce) at 4°C. Lysates were loaded onto SDS-PAGE gels followed by blotting onto PVDF membranes as described (Iwai et al., 2013). Blots were probed with primary antibodies followed by corresponding horseradish peroxidase-conjugated secondary antibodies. Primary antibodies include anti-PDGFRα #3174, CST; anti-pAKT (S473) #4058, CST; anti-AKT #4691, CST; anti-pERK-T202/Y204 #4370, CST; anti-ERK #9102, CST; anti-FGFR1 #76464, abcam; anti-BAF47 (SMARCB1) #61211, BD; anti-EGFR#2232, CST; anti-TFR #13-6890, ThermoFisherScientific; anti-pY1000 #8954, CST and anti-α-Tubulin #T5168, Sigma. Secondary antibodies include Polyclonal Goat Anti-Rabbit HRP #P0448, Dako and Anti-Mouse HRP #G32-62G-1000, Signalchem. Immunoreactive bands were visualized by chemiluminescence (Amersham) and the blots were exposed to x-ray XAR film (Kodak).

For immunoprecipitation, cells were lysed in RIPA lysis buffer (containing 1% Triton) supplemented with protease and phosphatase inhibitors (Thermo Pierce) at 4°C. After microcentrifugation at 2,000 rpm for 10 min, 200 µg of lysate was diluted in 200ul lysis buffer. Primary antibody (anti-PDGFR#3174, CST) was added at 1mg/ml and incubated with rotation overnight at 4 °C. Protein G plus agarose beads were added and incubated for three hours at 4 °C to collect immune complexes, washed five times with lysis buffer and eluted in sample buffer. Proteins were resolved by SDS–PAGE, transferred to PVDF membrane and immunoblotting was performed as described above.

For immunofluorescence experiments, cells were fixed with 4% formaldehyde for 15 min, permeabilised with 0.2% Triton-X 100/PBS for 10 min and then blocked with IF buffer (3% BSA, 0.05% Tween 20 in PBS) for 1h. Specimens were incubated overnight with primary antibodies (anti-PDGFR #3174, CST; anti-FGFR1 # PA5-18344, Thermo Fisher Scientific) at 4°C rinsed three times with IF buffer and then incubated with secondary antibodies (anti-rabbit Alexa488 and anti-goat Alexa555, Thermo Fisher Scientific). DNA was visualised by DAPI staining. Images were captured using a Zeiss 710 Confocal Microscope.

Cell viability and apoptosis assays

For Annexin V staining, 3000 cells/well were seeded into 96-well CellCarrier plates (Perkin Elmer). 24h after seeding, drugs were added and incubated for an additional 48h. Drugs that were used in this study include Dasatinib, Pazopanib, Sunitinib, Ponatinib, AZD4547, BEZ235 and Trametinib from LC laboratories; BGJ398 from Selleck Chemicals. FITC-Annexin V (BD Biosciences) and Hoechst 33342 (Tocris) diluted in 10x annexin binding buffer (0.1M HEPES,

1.4M NaCl, 25mM $CaCl_2$) was added and incubated at 37°C for 15 minutes. Plates were imaged using an Operetta high-content imager (Perkin Elmer). Images were analysed using Harmony software (Perkin Elmer), and annexin positivity defined as number of annexin-FITC-positive cells relative to total number of Hoechst-positive nuclei. The interaction between drugs was analysed by the Chou and Talalay median effect principle as described (Todd et al., 2014).

siRNA transfections were performed as follows, 2000 cells/well were reverse transfected in 96-well plates with SMARTpool siRNAs (Dharmacon) using Lullaby reagent (Oz Biosciences). Where indicated, cells were treated with vehicle or drug 24h post transfection. Apoptosis and cell viability were measured using Caspase 3/7 Glo and Cell Titre Glo (Promega), respectively, 72-96h post transfection according to manufacturer's instructions and normalised to cells transfected with a non-targeting siRNA pool.

aCGH, gene expression and phosphoproteomic analysis

For aCGH analysis, genomic DNA was extracted as previously described (Marchio et al., 2008; Natrajan et al., 2009). The aCGH platform was constructed in-house and comprises ~32,000 BAC clones tiled across the genome. This platform has been shown to be as robust as, and to have comparable resolution with, high-density oligonucleotide arrays (Coe et al., 2007; Gunnarsson et al., 2008). aCGH data were pre-processed and analyzed using the Base.R script in R version 2.14.0, as previously described (Natrajan et al., 2014). Genomic DNA from each sample was hybridized against a pool of normal female DNA derived from peripheral blood. Raw Log_2 ratios of intensity between samples and pooled female genomic DNA were read without background subtraction and normalized in the LIMMA package in R using PrinTipLoess. Outliers were removed based upon their deviation from neighboring genomic probes, using an estimation of the genome-wide median absolute deviation of all probes. Log_2 ratios were rescaled using the genome wide median absolute deviation in each sample and then smoothed using circular binary segmentation (cbs) in the DNACopy package as described (Natrajan et al., 2009). After filtering polymorphic BACs and BACs mapping to chromosome Y, a final dataset of 31,157 clones with unambiguous mapping information according to build hg19 of the human genome (http://www.ensembl.org). A categorical analysis was applied to the BACs after classifying them as representing amplification (>0.45), gain (>0.08 and ≤0.45), loss (<−0.08) or no change, according to their cbs-smoothed log2 ratio values (Marchio et al., 2008; Natrajan et al., 2009). Threshold values were determined and validated as previously described (Natrajan et al., 2009).

The Illumina Bead Chip (HumanHG-12 v4) data were pre-processed, log2-transformed, and quantile normalized using the *beadarray* package in Bioconductor (Dunning et al., 2007). We performed hierarchical clustering of the data using the MATLAB bioinformatics toolbox with Euclidean distance metric and average linkage to generate the hierarchical tree. Data rows (genes) were normalized so that the mean was 0 and the standard deviation was 1. Gene expression data has been deposited into the GEO repository, accession number GSE78864. Gene Set Enrichment Analysis (GSEA) software was used to identify the 50 genes with the strongest positive correlations and 50 genes with the strongest negative correlations to the phenotype (i.e. drug resistance). We ranked genes using the Signal2Noise (default) option in GSEA. Signal2Noise uses the difference of means scaled by the standard deviation for ranking defined as (mean1-mean2)/(st.dev1 + st.dev2). Statistical significance of gene correlations to the drug resistance phenotype (top 100 genes) were determined using the False Discovery Rate (FDR) method which corrects for multiple comparisons.

Phosphotyrosine proteomic analysis was performed as previously described (Iwai et al., 2013) with the following modifications: SILAC labelled cells (biological triplicates) were lysed in 8M urea and equal amounts of heavy (DasR or PasR cells) and light (parental cells) lysates were mixed prior to reduction, alkylation and trypsin digestion. Peptides were desalted on a C18 cartridge, eluted with 25% acetonitrile and lyophilised to dryness. A two-step enrichment of phosphotyrosine peptides was performed; immunoprecipitation (IP) using a combination of pTyr100, pTyr1000 (Cell Signalling) and 4G10 (Millipore) followed by immobilized metal affinity chromatography (IMAC) on $FeCl_3$ charged NTA beads as previously described (Iwai et al 2013). Eluted peptides were then subjected to reverse-phase liquid chromatography separation (Iwai et al 2013) followed by electrospray ionization and MS/MS on a Triple-TOF 5600+ mass spectrometer (ABSciex) operated in a data-dependent acquisition mode with top 25 most intense peaks (two to five positive charges) automatically acquired with previously selected peaks excluded for 30s. Detailed MS data related to Figure 2C is available upon request.

The data were processed with MaxQuant (Cox and Mann, 2008)(version 1.5.2.8) and the peptides were identified (maximal mass error = 0.006 Da and 40 ppm for precursor and product ions, respectively) from the MS/MS spectra searched against human referenced proteome (UniProt, June 2015) using Andromeda (Cox et al., 2011) search engine. The following peptide bond cleavages: arginine or lysine followed by any amino acid (a general setting referred to as Trypsin/P) and up to two missed cleavages were allowed. SILAC based experiments in MaxQuant were performed using the built-in quantification algorithm (Cox and Mann 2008) with minimal ratio count = 2 and enabled 'Re-quantify' feature. Cysteine carbamidomethylation was selected as a fixed modification whereas methionine oxidation, acetylation of protein N-terminus and phospho (STY) as variable modifications. The false discovery rate was set to 0.01

for peptides, proteins and sites. Other parameters were used as pre-set in the software. "Unique and razor peptides" mode was selected to allow identification and quantification of proteins in groups.

Data were further analysed using Microsoft Office Excel 2007 and Perseus (version 1.5.0.9). The data were filtered to remove potential contaminants and IDs originating from reverse decoy sequences. The log2 values of the heavy/light (H/L) ratios were then determined. An arbitrary value of +10 or -10 was manually imputed when only H or L intensity, respectively, was detected and thus the H/L ratio could not have been automatically assigned by MaxQuant. The data were then normalized to the average H/L ratio of the total proteome (IP supernatant) and filtered to include only high confidence phosphosite IDs (localization probability and score difference ≥ 90% and 10, respectively). For generation of the heat map (Figure 2C), normalized H/L ratios of respective triplicates were averaged and reversed (L/H) to visualize the log2 fold changes in phosphorylation between parental (L) and resistant (H) cells.

RNA Seq and immunohistochemistry analysis of patient specimens

RNA was extracted using a Qiagen all-prep kit from 23 fresh frozen tumour tissue samples taken from paediatric patients with a confirmed diagnosis of SMARCB1 negative MRT. A paired end cDNA sequencing library was created using Illumina's Tru-seqv2 RNA library preparation kit according to manufacturer's instructions. Libraries were sequenced on an Illumina Hi-Seq2500 to give ~90M paired end reads. Reads were QC checked (FastQC) and aligned using RNA-STAR (Dobin et al., 2013), and gene counts generated using Gencodev19 Transcriptome library and HTSeq-count (Anders et al., 2015). Library normalisation, dispersion estimations and variance stabilising transformations were generated using R Bioconductor package DESeq2 (Love et al., 2014) as were adjusted P-values and moderated log fold change values calculated by one-sided t-test to indicate significant differences in expression between RT samples and the normal tissue collection. RNA-seq data from normal tissues were taken from Illumina Bodymap (ArrayExpress E-MTAB-513, E-MTAB-2836). Variance stabilised RNASeq data is provided in Table S3.

The fully automated Ventana BenchMark XT IHC system and standard detection reagents ultraVIEW™ Universal DAB Detection kit (Ventana Medical Systems; Cat No: 760-500) were used to demonstrate SMARCB1 (BAF47 BD Transduction Labs Cat No: 612111) in 4 micrometre tissue sections at a dilution of 1/100. This incorporated antigen retrieval with Ventana ultra cell conditioning 1 (64 minutes) and a haematoxylin counterstain (Ventana Medical Systems: Cat No: 950-224). PDGFRα and FGFR1 were both demonstrated using the Dako Autostainer Link48 platform. Briefly, slides were antigen retrieved using pH6 citrate, either via microwave (PDGFRα) or MenaPath Access Retrieval Unit (FGFR1) before being placed onto the autostainer where they were incubated in primary antibody, diluted 1/250 for PDGFRα (3714 Cell Signalling) and 1/50 for FGFR1 (ab76464 Abcam) and detected using Dako EnVision FLEX reagents (Dako K8002).

Supplemental References

Anders, S., Pyl, P.T., and Huber, W. (2015). HTSeq--a Python framework to work with high-throughput sequencing data. Bioinformatics *31*, 166-169.

Coe, B.P., Ylstra, B., Carvalho, B., Meijer, G.A., Macaulay, C., and Lam, W.L. (2007). Resolving the resolution of array CGH. Genomics *89*, 647-653.

Cox, J., and Mann, M. (2008). MaxQuant enables high peptide identification rates, individualized p.p.b.-range mass accuracies and proteome-wide protein quantification. Nat Biotechnol *26*, 1367-1372.

Cox, J., Neuhauser, N., Michalski, A., Scheltema, R.A., Olsen, J.V., and Mann, M. (2011). Andromeda: a peptide search engine integrated into the MaxQuant environment. J Proteome Res *10*, 1794-1805.

Dobin, A., Davis, C.A., Schlesinger, F., Drenkow, J., Zaleski, C., Jha, S., Batut, P., Chaisson, M., and Gingeras, T.R. (2013). STAR: ultrafast universal RNA-seq aligner. Bioinformatics *29*, 15-21.

Dunning, M.J., Smith, M.L., Ritchie, M.E., and Tavare, S. (2007). beadarray: R classes and methods for Illumina bead-based data. Bioinformatics *23*, 2183-2184.

Gunnarsson, R., Staaf, J., Jansson, M., Ottesen, A.M., Goransson, H., Liljedahl, U., Ralfkiaer, U., Mansouri, M., Buhl, A.M., Smedby, K.E., *et al.* (2008). Screening for copy-number alterations and loss of heterozygosity in chronic lymphocytic leukemia--a comparative study of four differently designed, high resolution microarray platforms. Genes Chromosomes Cancer *47*, 697-711.

Iwai, L.K., Payne, L.S., Luczynski, M.T., Chang, F., Xu, H., Clinton, R.W., Paul, A., Esposito, E.A., Gridley, S., Leitinger, B., *et al.* (2013). Phosphoproteomics of collagen receptor networks reveals SHP-2 phosphorylation downstream of wild-type DDR2 and its lung cancer mutants. Biochem J *454*, 501-513.

Love, M.I., Huber, W., and Anders, S. (2014). Moderated estimation of fold change and dispersion for RNA-seq data with DESeq2. Genome Biol *15*, 550.

Marchio, C., Iravani, M., Natrajan, R., Lambros, M.B., Savage, K., Tamber, N., Fenwick, K., Mackay, A., Senetta, R., Di Palma, S., *et al.* (2008). Genomic and immunophenotypical characterization of pure micropapillary carcinomas of the breast. J Pathol *215*, 398-410.

Natrajan, R., Lambros, M.B., Rodriguez-Pinilla, S.M., Moreno-Bueno, G., Tan, D.S., Marchio, C., Vatcheva, R., Rayter, S., Mahler-Araujo, B., Fulford, L.G., *et al.* (2009). Tiling path genomic profiling of grade 3 invasive ductal breast cancers. Clin Cancer Res *15*, 2711-2722.

Natrajan, R., Wilkerson, P.M., Marchio, C., Piscuoglio, S., Ng, C.K., Wai, P., Lambros, M.B., Samartzis, E.P., Dedes, K.J., Frankum, J., *et al.* (2014). Characterization of the genomic features and expressed fusion genes in micropapillary carcinomas of the breast. J Pathol *232*, 553-565.

Todd, J.R., Scurr, L.L., Becker, T.M., Kefford, R.F., and Rizos, H. (2014). The MAPK pathway functions as a redundant survival signal that reinforces the PI3K cascade in c-Kit mutant melanoma. Oncogene *33*, 236-245.
